# CRY1 interacts directly with HBI1 to regulate its transcriptional activity and photomorphogenesis in Arabidopsis

**DOI:** 10.1093/jxb/ery209

**Published:** 2018-06-01

**Authors:** Sheng Wang, Ling Li, Pengbo Xu, Hongli Lian, Wenxiu Wang, Feng Xu, Zhilei Mao, Ting Zhang, Hongquan Yang

**Affiliations:** 1 School of Life Sciences and Biotechnology, Shanghai Jiaotong University, Shanghai, China; 2 School of Agriculture and Biology, Shanghai Jiaotong University, Shanghai, China; 3 State Key laboratory of Genetic Engineering, Collaborative Innovation Center for Genetics and Development, School of Life Sciences, Fudan University, Shanghai, China

**Keywords:** Arabidopsis, blue light, cell elongation, cryptochrome, HBI1, protein interaction, transcriptional activity

## Abstract

Cryptochromes (CRYs) are blue light photoreceptors that mediate various light responses in plants and animals. In Arabidopsis, there are two homologous CRYs, CRY1 and CRY2, which mediate blue light inhibition of hypocotyl elongation. It is known that CRY2 interacts with CIB1, a basic helix–loop–helix (bHLH) transcriptional factor, to regulate transcription and floral induction. In this study, we performed yeast two-hybrid screening and identified CIB1 as a CRY1-interacting protein. Moreover, we demonstrated that CRY1 physically interacted with the close homolog of CIB1, HBI1, which is known to act downstream of brassinosteroid (BR) and gibberellin acid (GA) signaling pathways to promote hypocotyl elongation, in a blue light-dependent manner. Transgenic and genetic interaction studies showed that overexpression of HBI1 resulted in enhanced hypocotyl elongation under blue light and that HBI1 acted downstream of CRYs to promote hypocotyl elongation. Genome-wide gene expression analysis indicated that CRYs and HBI1 antagonistically regulated the expression of genes involved in regulating cell elongation. Moreover, we demonstrated that CRY1–HBI1 interaction led to inhibition of HBI1’s DNA binding activity and its target gene expression. Together, our results suggest that HBI1 acts as a new CRY1-interacting protein and that the signaling mechanism of CRY1 involves repression of HBI1 transcriptional activity by direct CRY1–HBI1 interaction.

## Teaser

CRY1 interacts with HBI1 in a blue light-dependent manner. CRY1 and HBI1 antagonistically regulate expression of genes promoting cell elongation and CRY1 represses HBI1’s transcriptional activity by inhibiting its DNA binding ability.

## Introduction

Plants on the earth are influenced by many kinds of environmental factors during their life cycle from seed germination to flowering, among which light provides not only the essential source of energy for photosynthesis but also regulatory signals for growth and development. Plants have evolved multiple photoreceptors to perceive light quality, quantity, and direction, which include the UV-B light photoreceptor UVR8, the blue light photoreceptors cryptochromes (CRYs) and phototropins, and the red/far-red light photoreceptors phytochromes (Cashmore *et al.*, 1999; Briggs and Christie, 2002; Quail, 2002; Rizzini *et al.*, 2011). Among these photoreceptors, cryptochromes play critical roles not only in plants, but also in *Drosophila* and mammals. In Arabidopsis, there are two well-studied homologous CRYs, CRY1 and CRY2, which play relatively major roles in regulating photomorphogenesis under blue light and photoperiodic flowering, respectively (Ahmad and Cashmore, 1993; Guo *et al.*, 1998; Lin *et al.*, 1998). Photomorphogenesis is one of the critical and best studied light-controlled processes in Arabidopsis, which is characterized by shortened hypocotyls, expanded cotyledons, and accumulation of chlorophyll under light, and cryptochromes and phytochromes are shown to play critical roles in regulating this process (Pepper *et al.*, 1994; McNellis and Deng, 1995; Cashmore *et al.*, 1999; Deng and Quail, 1999; Quail, 2002; Lin and Shalitin, 2003). Furthermore, both CRY1 and CRY2 have been shown to entrain the circadian clock and mediate blue light induction of stomatal opening and development (Somers *et al.*, 1998; Mao *et al.*, 2005; Kang *et al.*, 2009). In *Drosophila*, cryptochrome acts in the input pathway to entrain the circadian clock (Emery *et al.*, 1998) and, in mammals, cryptochromes serve as integral components of the central oscillator of the circadian clock (Kume *et al.*, 1999). In migratory birds, cryptochromes act as the magnetoreceptors to perceive magnetic fields and navigate during long-distance migration (Gegear *et al.*, 2010).

Cryptochomes are structurally divided into an N-terminal domain that shares high sequence similarity to photolyases, and a distinguishing C-terminal extension that is absent in photolyases (Sancar, 1994; Cashmore *et al.*, 1999; Lin and Shalitin, 2003). It has been shown that the C-terminal domains of Arabidopsis CRY1 and CRY2 mediate CRY1/2 signaling through direct interactions with COP1 (Yang *et al.*, 2000, 2001; Wang *et al.*, 2001), a RING-finger E3 ubiquitin ligase (Deng *et al.*, 1992) that interacts with and targets the degradation of a set of transcription factors, such as HY5/HYH and CONSTANS (CO), to regulate photomorphogenesis and flowering under long days, respectively (Putterill *et al.*, 1995; Oyama *et al.*, 1997; Osterlund *et al.*, 2000; Lee *et al.*, 2007; Jang *et al.*, 2008; L.J. Liu *et al.*, 2008). Moreover, CRY1 and CRY2 also interact with the COP1 enhancer, SPA1 (Lian *et al.*, 2011; Liu *et al.*, 2011; Zuo *et al.*, 2011), which interacts with COP1 to enhance its E3 ligase activity (Seo *et al.*, 2003). The outcome of interactions of CRY1/CRY2 with COP1/SPA1 is disruption of the COP1–SPA1 core complex, thus stabilizing HY5/CO proteins and promoting their accumulation. The N-terminal domain of CRY1 and CRY2 mediate CRY dimerization (Sang *et al.*, 2005; Yu *et al.*, 2007), and it is shown that CRY2 dimerization is inhibited by Blue-light Inhibitor of Cryptochromes 1 (BIC1) (Q. Wang *et al.*, 2016). Recently, the CRY1 N-terminus has been shown to mediate CRY1 signaling independent of the CRY1 C-terminus (He *et al.*, 2015). However, the underlying mechnism is not well understood.

The discovery showing direct cryptochrome regulation of transcription comes from screening for CRY2-interacting factors, which leads to the identification of CIB1 (cryptochrome interacting basic helix–loop–helix1). It has been shown that CRY2 interacts with CIB1 through its N-terminus to regulate the transcriptional activity of CIB1 and floral initiation (H. Liu *et al.*, 2008). HBI1 (HOMOLOG OF BEE2 INTERACTING WITH IBH 1) is also a basic helix–loop–helix (bHLH) transcriptional factor that shows high amino acid sequence similarity to CIB1 and its homologs, CIL1 (CIB1 like protein 1) and BEE2 (BR enhanced expression 2) (Bai *et al.*, 2012*a*). Previous studies have demonstrated that HBI1, as well as CIBs (CIB4/5), CILs (CIL1/2) ,and BEEs (BEE1/2/3), have similar functions and work to promote hypocotyl elongation in Arabidopsis, respectively (Friedrichsen *et al.*, 2002; Bai *et al.*, 2012*a*; Ikeda *et al.*, 2012). It has been demonstrated that ILI1 BINDING bHLH PROTEIN1 (IBH1) interacts with HBI1 to inhibit HBI1 transcriptional activity and hypocotyl elongation by repressing the DNA binding ability of HBI1, and that the inhibitory effects of IBH1 on HBI1 transcriptional activity is counteracted by PRE1 (PACLOBTRAZOL RESISTANCE1) through its direct interaction with IBH1 (Bai *et al.*, 2012*a*). Whether CRY1 interacts with CIB1 and its homologs through its N-terminus to regulate hypocotyl elongation remains unknown.

In this study, we sought potential CRY1 N-terminus (CNT1)-interacting proteins through yeast two-hybrid screening, and identified CIB1 as a candidate. We confirmed by yeast two-hybrid and protein co-localization assays that CNT1 interacts with CIB1 and its homologs BEE2 and CIL1. Furthermore, we demonstrated by yeast two-hybrid, pull-down, protein co-localization, and co-immunoprecipitation (co-IP) assays that CRY1 interacted with HBI1 in a blue light-dependent manner. By making transgenic lines in which HBI1 function was either enhanced or repressed, we demonstrated that HBI1 acted to regulate hypocotyl elongation positively under blue, red, and far-red light, respectively. Moreover, by creating transgenic lines in which HBI1 function was suppressed in the *cry1cry2* mutant background, we demonstrated that HBI1 acted downstream of CRY1 and CRY2 to regulate hypocotyl elongation under blue light. We demonstrated by genome-wide gene expression studies that CRY1/2 and HBI1 acted antagonistically to regulate the expression of a set of genes related to cell elongation. We also showed by EMSA and dual luciferase (Dual-LUC) assays that CRY1 inhibited the transcriptional activity of HBI1 through its N-terminus. This study therefore reveals a new mechanism of CRY1 signaling, which involves direct blue light-dependent interaction with the transcriptional factor HBI1 to regulate its transcriptional activity and photomorphogenesis.

## Materials and methods

### Plant materials and growth conditions

*Arabidopsis thaliana* ecotype Columbia (Col-0) was used as the wild-type (WT) control. *cry1cry2*, *phyA-211*, and *phyB-9* mutants, and transgenic lines overexpressing Myc-CRY1 (*CRYI-OX*), Myc-CRY2 (*CRY2-OX*), and *GUS-CCT1* (*GUS-CCT1*) were described previously (Yang *et al.*, 2000; Mao *et al.*, 2005; Sang *et al.*, 2005). HBI1-related transgenic plants were generated using corresponding constructs by an *in planta* method (Bechtold and Pelletier, 1998). For phenotype analyses of seedlings, seeds were sown on half-strength Murashige and Skoog (MS) medium plus 1% sucrose with 0.8% agar and were cold treated for 4 d at 4 °C. After exposure to white light for 12 h to promote germination, plates with seeds were transferred to appropriate light conditions for 4 d at 22 °C. For adult plants, seeds were sown as above and grown under continuous white light for 7 d, then transferred to soil to continue growing for the time indicated.

### Plasmid construction

The constructs expressing COP1 in yeast, and cLUC-Flag, CRY1–yellow fluorescent protein (YFP), CRY2–YFP, β-glucuronidase (GUS)–nuclear localization signal (NLS)–YFP, and CNT1–NLS–YFP in plants were described previously (Lian *et al.*, 2011; He *et al.*, 2015; Xu *et al.*, 2016). PCR-amplified fragments encoding HBI1, HBI1– ERF-associated amphiphilic repression (EAR), CNT1–NLS, NLS–CCT1, CIB1, CNT2–NLS, NLS–CCT2, CRY2, BEE2, CIL1, CO, and NLS–GUS–CCT1 were cloned into the multiple cloning sites (MCS) of pHB-*MCS-3×Flag*, pHB-*6×Myc-MCS*, pGBKT7, pGADT7, pHB-*MCS*-YFP [cyan fluorescent protein (CFP)], and pHB-YFP (CFP)-*MCS*, respectively. All of the constructs and primers used in this study are listed in Supplementary Table S1 at *JXB* online.

### Analyses of RNA-Seq data

The RNA sequencing (RNA-Seq) data indicating the genes regulated by HBI1 and CRYs have the accession numbers GSE53078 and GSE58552, respectively (Fan *et al.*, 2014; He *et al.*, 2015). A Venn diagram was generated in Venny (http://bioinfogp.cnb.csic.es/tools/venny/index.html). A heatmap was generated with hierarchical clustering analysis by the MeV 4.7 software. Gene Ontology (GO) analysis was performed in agriGO v2.0 (http://systemsbiology.cau.edu.cn/agriGOv2/) (Tian *et al*., 2017), with TAIR10 being as the reference and hypergeometric statistical test method and complete GO type.

### Quantitative real-time PCR

We used WT, *cry1cry2*, *CRY1-OX*, *CRY2-OX*, *HBI1-EAR-OX/cry1cry2*, and *HBI1-OX* seedlings for quantitative real-time PCR (qRT-PCR). Seeds were sown as above and then subjected to white light treatment for 12 h to promote germination. The plates with seeds were kept in darkness for 4 d before being exposed to blue light irradiation. Total RNAs were isolated according to the manufacturer’s instructions (TIANGEN). The detailed method of qRT-PCR was described previously (Zhang *et al.* 2014). *PP2A* was used as an internal control. The primers used are listed in Supplementary Table S1.

### Yeast two-hybrid assay

Yeast two-hybrid assay was performed according to the user manual for the Yeastmaker Yeast Transformation System (Clontech Laboratories, Mountain View, CA, USA). Combinations containing the fragments fused in pGBKT7 and pGADT7 vectors were co-transformed into the AH109 strain via the PEG/LiAc transformation procedure. At 3 d after transformation, yeast cells were spread on selective media with 3-amino-1,2,4-triazole (3AT) in continuous blue light or darkness for 3 d.

### Pull-down assay

For *in vitro* pull-down assay, the fragments encoding CNT1, CCT1, and HBI1 were each cloned into pCold-trigger factor (TF) or pMAL-c2X vector. The fusion proteins were expressed in *Escherichia coli* (Rosetta) and purified according to the manufacturer’s protocol (QIAGEN and NEB). Maltose-binding protein (MBP)–HBI1 was first incubated with amylose–magnetic beads (NEB) in TBST buffer (50 mM Tris–HCl, pH 7.5, 150 mM NaCl, 0.2% Triton X-100, and 10% glycerol) containing 1 mM proteinase inhibitor (Pefabloc, Roche) at 4 °C for 4 h, then the amylose–magnetic beads affiliated with MBP–HBI1 serving as bait was washed with TBST three times and incubated with the same amounts (~0.2 μg) of prey proteins of His-TF, His-TF–CNT1, and His-TF–CCT1 in a final volume of 1 ml at 4 °C for 0.5 h. Then the beads were washed with TBST three times, dissolved in 30 μl of 2× SDS loading buffer, and subjected to western blot or Coomassie Brilliant Blue staining.

For the semi-*in vivo* pull-down assay with Arabidopsis protein extracts, *CRY1-OX* seedlings grown in soil for ~2 weeks under white light were kept in darkness for 2 d before they were transferred to red light (30 μmol m^–2^ s^–1^), blue light (50 μmol m^–2^ s^–1^), far-red light (10 μmol m^–2^ s^–1^), or continuing darkness for 3 h. These seedlings were quickly frozen by liquid nitrogen and homogenized with TBST buffer containing 1 mM proteinase inhibitor (Pefabloc and Cocktail, Roche). The supernatant adjusted to contain the same amount of total protein quantified by Bradford assay (Biorad) was infused to tubes containing MBP–HBI1-conjugated beads, incubated for 30 min at 4 °C, and washed three times with 1 ml of TBST buffer each time, then the precipitates were eluted into 30 μl of 2× SDS loading buffer and subjected to western blot analysis with anti-Myc antibody (Millipore).

### Protein co-localization assay

*Agrobacterium tumefaciens* strain GV3101 harboring the constructs expressing the studied proteins or p19 plasmid were diluted by MS liquid medium to OD_600_=0.6 individually and treated with 200 μM acetosyringone and 10 mM MES (pH 5.6) for 3 h at room temperature. Then the mixture of *Agrobacterium* harboring the constructs expressing CFP and YFP fusion proteins, and p19 plasmid with a volume ratio of 1:1:1 were introduced into tobacco (*Nicotiana benthamiana*) leaf epidermal cells by inﬁltration. After incubation for 2 d or 3 d in dim light, leaves were collected for confocal microscopic examination (Leica TCS SP5II confocal laser scanning microscope).

### Co-immunoprecipitation assay

Tobacco leaves were inﬁltrated with a mixture of *Agrobacterium* culture (OD_600_=0.6) harboring the constructs expressing p19, HBI1-Flag, and YFP fusion proteins or p19, CNT1–NLS–YFP, and Flag-tagged fusion proteins, with a volume ratio of 1:1:1 and then kept in dim light for 3 d as for co-localization assays. Samples from tobacco leaves were collected and homogenized in TBST containing proteinase inhibitors. After centrifugation, protein supernatant was incubated with 10 μl of protein G magnetic beads (Invitrogen) [which had been incubated with 1 μl of anti-Flag antibody (Sigma) overnight at 4 °C] for 1 h at 4 °C. The immunoprecipitate was washed three times with TBST and eluted with 0.5 μg μl^–1^ 3×Flag peptide (Sigma) in 30 μl of TBST. A 24 μl aliquot of eluate was resuspended with 6 μl of 5× SDS loading buffer, boiled for 5 min, and then subjected to immunoblot analysis with anti-Flag (Sigma) and anti-green fluorescent protein (GFP; Abmart) antibodies.

### Hypocotyl measurement

Seedlings were photographed, and then the digital photographs were utilized to measure hypocotyl length through Image J software (http://rsbweb.nih.gov/ij/).

### Western blotting

Arabidopsis seeds of different genotypes were grown on half-strength MS medium under continuous white light for 5 d. Seedlings with different light treatments were collected and homogenized in TBST described above, and the protein supernatant, after quantification by Bradford assay (BioRad), was subjected to immunoblot analysis with the related antibody.

### EMSA

The proteins used in this experiment were expressed and purified as described above prior to being quantified by comparison with the BSA standard, then 1.5 μg of MBP and 0.5 μg of MBP–HBI1 were utilized. Biotin-labeled probes were commercially synthesized and annealed. The binding reaction was carried out in 20 μl of reaction buffer [10× Binding buffer, 100 mM MgCl_2_, 1 μg μl^–1^ poly(dI dC), 1% NP-40] with 20 fM probe and purified proteins according to the manufacturer’s instructions (Thermo Scientific). The reactions were resolved by 6% native PAGE at 4 °C after 15 min incubation at room temperature, and then the DNA–protein complexes were transferred to a nylon membrane. Biotin-labeled probes were detected by horseradish peroxidase (HRP)-conjugated streptavidin and visualized with an ECL detection kit.

### Dual-LUC assay

An ~2 kb fragment upstream of the start codon of *Exp16* was amplified and cloned into pGreen0800 vector, which expresses Renilla luciferase (REN) driven by the 35S promoter serving as an internal reference and firefly luciferase (LUC) driven by the *Exp16* promoter serving as a reporter. *Agrobacterium* culture (OD_600_=0.6) harboring the related constructs was mixed with the volume ratio indicated in Fig. 8F, G and Supplementary Fig. S7; the lack of any effector in all groups was supplemented with liquid MS medium to the same volume. The mixture was introduced into tobacco leaves by inﬁltration and kept in dim white light for 3 d, and then the samples were collected for measurements of luciferase activity using commercial reagents according to the manufacturer’s instructions (Promega).

## Results

### The CRY1 N-terminus interacts with CIB1 and its homologs BEE2 and CIL1 in yeast and tobacco cells

**Fig. 1.**
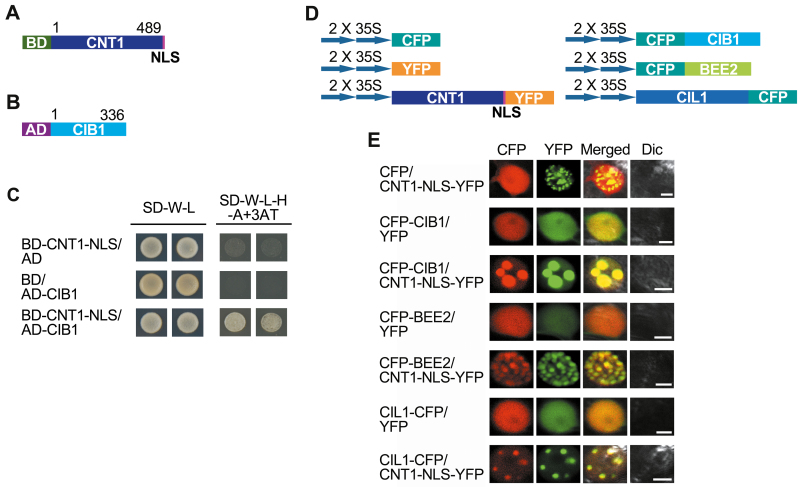
CNT1 physically interacts with CIB1, BEE2, and CIL1 in yeast and tobacco cells. (A) Bait protein in yeast two-hybrid assay. CNT1 is fused with the GAL4-binding domain (BD) and nuclear localization signal (NLS) sequence. (B) Prey protein in yeast two-hybrid assay. CIB1 is fused with the GAL4 activation domain (AD). (C) Analysis of CNT1–CIB1 interaction in yeast cells. In this and other figures, all vector combinations are given as bait/prey. Yeast cells co-expressing CNT1/CIB1 were grown on basic (SD-W-L) or selective media with 10 mM 3AT (SD-W-L-H-A+3AT) in darkness for 3 d. (D) Schematic diagram of constructs expressing CNT1 fused to the NLS sequence and yellow ﬂuorescent protein (YFP) and constructs expressing CIB1, BEE2, and CIL1 fused to cyan ﬂuorescent protein (CFP). (E) Co-localization assay showing that CNT1 and these homologous proteins, CIB1, BEE2, and CIL1, localize together to the same nuclear bodies (NBs) in tobacco cells. Dic, differential interference contrast. Scale bars=5 μm.

Our previous demonstration that CNT1 is able to mediate CRY1 signaling independent of its C-terminus (He *et al.*, 2015) prompted us to look for potential CNT1-interacting proteins. To this end, we carried out GAL4 yeast two-hybrid screening against CNT1 and identified 15 clones encoding CIB1, a bHLH transcriptional factor with which CRY2 interacts through its N-terminus to regulate photoperiodic flowering (H. Liu *et al.*, 2008). To confirm the interaction of CNT1 with CIB1, we performed GAL4 yeast two-hybrid assays by co-transforming yeast cells with the bait construct expressing CNT1 fused to the GAL4 DNA-binding domain (BD) tagged by an NLS sequence (Fig. 1A), together with a prey construct expressing the GAL4 activation domain (AD) fused to CIB1 (Fig. 1B). The results showed that CNT1 interacted with CIB1 (Fig. 1C). We then performed an *in vivo* protein co-localization assay by making constructs expressing CNT1 fused to the NLS followed by YFP, CIB1, and its homologs BEE2 and CIL1 fused to cyan CFP (Fig. 1D). As shown in Fig. 1E, expression of CNT1–NLS–YFP produced nuclear bodies (NBs) in tobacco leaf epidermal cells, whereas expression of CFP–CIB1, CFP–BEE2, or CIL1–CFP did not. However, when co-expressed with CNT1–NLS–YFP, CFP–CIB1, CFP–BEE2, and CIL1–CFP proteins were co-localized to the same NBs as CNT1–NLS–YFP, indicating interactions of CNT1 with these proteins.

### CNT1 physically interacts with HBI1 in yeast cells and *in vitro*

**Fig. 2.**
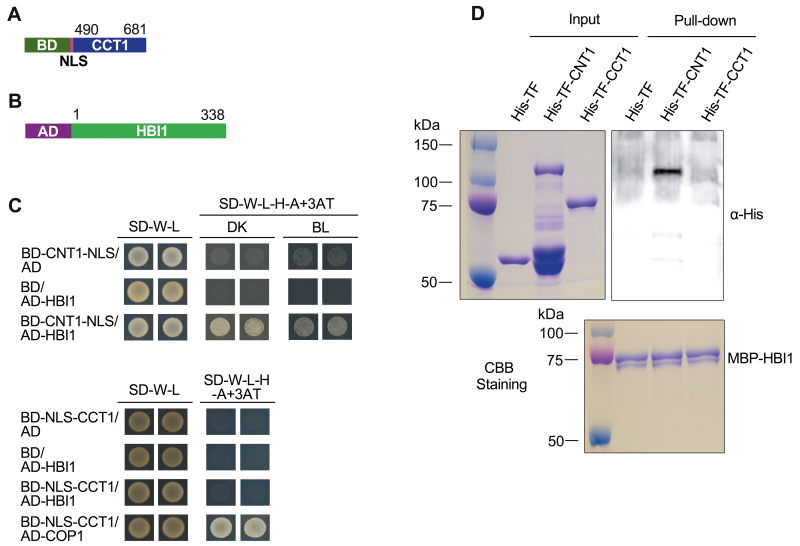
CNT1 physically interacts with HBI1 in yeast cells and *in vitro*. (A) Bait protein. CCT1 is fused with the GAL4-binding domain (BD) and NLS sequence. (B) Prey protein. HBI1 is fused with the GAL4 activation domain (AD). (C) HBI1 interacts with CNT1 rather than CCT1 in yeast cells. Yeast cells co-expressing CNT1/HBI1 were grown on basic (SD-W-L) or selective media with 10 mM 3AT (SD-W-L-H-A+3AT) in continuous 30 μmol m^–2^ s^–1^ blue light (BL) or darkness (DK) for 3 d at the top or cells co-expressing CCT1/HBI1 were grown on basic (SD-W-L) or selective media with 2 mM 3AT (SD-W-L-H-A+3AT) in darkness for 3 d at the bottom. (D) MBP pull-down assay showing the interaction of HBI1 with CNT1 rather than CCT1. CBB Staining denotes Coomassie Brilliant Blue staining. His-TF was used as negative control. All these experiments were independently repeated three times.

Among the CIBs and CIB-like proteins, HBI1 is preferentially expressed in hypocotyl and cotyledon, and acts as a positive regulator of cell elongation downstream of the BR (brassinosteroid) and GA (gibberellin acid) signaling pathways through the PREs (PACLOBTRAZOL RESISTANCE1)–IBH1 (ILI1 BINDING bHLH PROTEIN1)–HBI1 module (Bai *et al.* 2012*a*). We therefore focused on exploring whether CRY1 might interact directly with HBI1 through CNT1 to regulate hypocotyl elongation. To test this possibility, we first performed a yeast two-hybrid assay with bait constructs expressing CNT1–NLS (Fig. 1A) and the CRY1 C-terminus fused to an NLS (NLS–CCT1) (Fig. 2A), and prey constructs expressing the full-length HBI1 (Fig. 2B) and COP1 (Lian *et al.*, 2011), respectively. As shown in Fig. 2C, CNT1 interacted with HBI1 in both darkness and blue light, whereas CCT1 did not interact with HBI1 in either darkness or blue light but interacted with the positive control, COP1 (Yang *et al.*, 2001). These results indicated that CNT1 interacted with HBI1 in yeast cells. We then performed an *in vitro* pull-down assay to confirm the interaction of CNT1 with HBI1 using CNT1 and CCT1 polypeptides tagged by His-tagged TF, and HBI1 protein tagged by MBP expressed in *E*. *coli*. The results showed that CNT1 was pulled-down by HBI1, but CCT1 was not (Fig. 2D). These results indicate that CNT1 rather than CCT1 might mediate the interaction of CRY1 with HBI1.

### CRY1 interacts with HBI1 through its N-terminus in plant cells

**Fig. 3.**
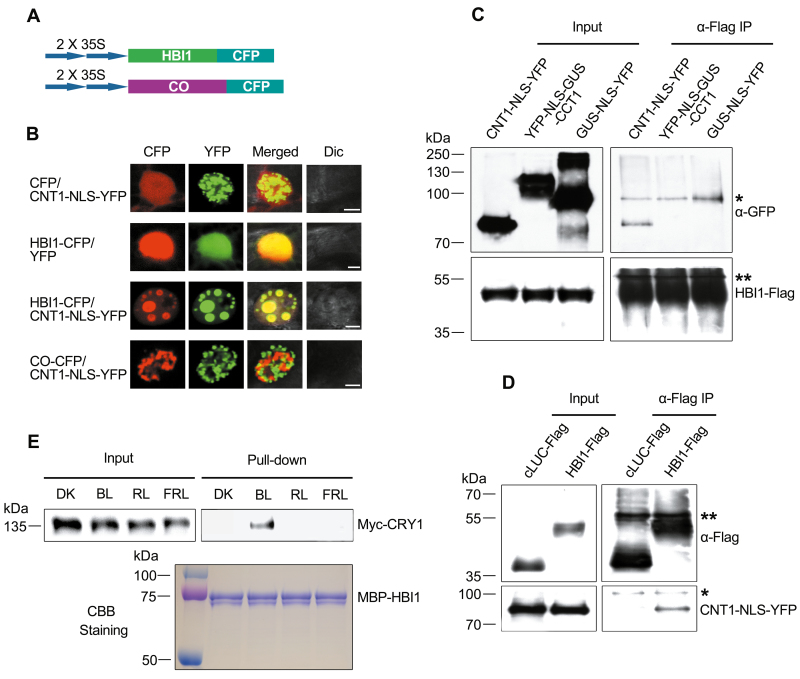
CRY1 physically interacts with HBI1 through its N-terminus in a blue light-dependent manner. (A) Schematic diagram of constructs expressing HBI1 and the negative control CO fused to CFP. (B) Co-localization assay of studied proteins in tobacco cells. CNT1 and HBI1 localize together to the same NBs, whereas CNT1 and CO localize to different NBs. Dic, differential interference contrast. Scale bars=5 μm. (C) Co-IP assay showing interaction of CNT1 with HBI1. YFP-tagged CNT1, CCT1, and the negative control, GUS protein, were co-expressed with Flag-tagged HBI1 in tobacco leaves. The immunoprecipitates were detected by anti-GFP and anti-Flag antibodies. A single asterisk and double asterisks denote a non-specific band and the heavy chain of IgG, respectively. (D) Co-IP assay showing that CNT1 interacts specifically with HBI1. Flag-tagged HBI1 and the negative control, cLUC protein, were co-expressed with YFP-tagged CNT1 in tobacco leaves. The immunoprecipitates were detected by anti-Flag and anti-GFP antibodies. A single asterisk and double asterisks denote a non-specific band and the heavy chain of IgG, respectively. (E) Semi-*in vivo* pull-down assay showing the blue light-specific interaction of CRY1 with HBI1. MBP–HBI1 served as bait. Myc-CRY1-containing protein extracts from *CRY1-OX* seedlings that were adapted to darkness (DK) or exposed to 50 μmol m^–2^ s^–1^ blue (BL), 30 μmol m^–2^ s^–1^ red (RL), or 10 μmol m^–2^ s^–1^ far-red (FRL) light for 3 h served as preys. CBB Staining denotes Coomassie Brilliant Blue staining. All experiments above were independently repeated at least three times.

With the demonstration that CNT1 interacts with HBI1 in yeast cells and in an *in vitro* pull-down assay, we asked whether CNT1 might interact with HBI1 *in vivo*. To test this possibility, we first performed a protein co-localization assay in tobacco cells using a construct expressing HBI1 tagged by CFP (Fig. 3A) and a construct expressing CNT1–NLS–YFP (Fig. 1D). The results showed that, CO–CFP (Fig. 3A) and CNT1–NLS–YFP expressed in the same tobacco cells were not merged together and localized to different NBs, while CNT1–NLS–YFP and HBI1–CFP proteins were co-localized to the same NBs (Fig. 3B), indicating a possible interaction of CNT1 with HBI1. Next, we performed a co-IP assay with tobacco leaves transiently co-expressing CNT1–NLS–YFP, YFP–NLS–GUS–CCT1, or GUS–NLS–YFP with Flag-tagged HBI1 proteins to confirm the interaction of CNT1 with HBI1. The results showed that CNT1 was immunoprecipitated by HBI1, but not by CCT1 or the control protein GUS (Fig. 3C), further indicating an interaction of CNT1 with HBI1. Moreover, co-IP assay with tobacco leaves transiently co-expressing Flag-tagged HBI1 or cLUC (the C-terminus of firefly luciferase) with CNT1–NLS–YFP confirmed that the binding of CNT1 to HBI1 is specific (Fig. 3D). Furthermore, we performed a semi-*in vivo* pull-down assay using MBP–HBI1 fusion protein as a bait, and Myc-CRY1-containing protein extracts as preys, which were prepared from Arabidopsis seedlings overexpressing Myc-CRY1 (*CRY1-OX*) adapted in the dark and exposed to blue, red, and far-red light. The results showed that MBP–HBI1 pulled-down Myc-CRY1 from *CRY1-OX* seedlings exposed to blue light, but not from those either adapted in darkness or exposed to red or far-red light (Fig. 3E). These results indicate that CRY1 interacts with HBI1 in a blue light-dependent manner.

### CRY2 interacts with HBI1 through its N-terminus

**Fig. 4.**
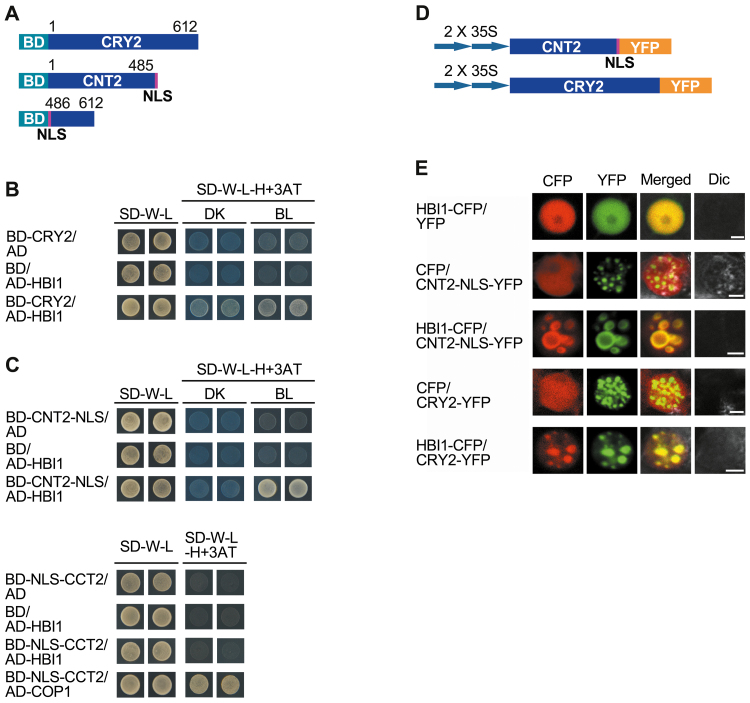
CRY2 interacts with HBI1 through its N-terminus in yeast and tobacco cells. (A) Bait proteins. All proteins, CNT2, CCT2, and CRY2, are fused with the GAL4 binding domain (BD), among which CNT2 and CCT2 are also tagged with the NLS sequence. (B) The physical interaction of CRY2 and HBI1 analyzed in yeast cells. Yeast cells co-expressing CRY2/HBI1 were grown on basic (SD-W-L) or selective media with 5 mM 3AT (SD-W-L-H+3AT) in continuous 30 μmol m^–2^ s^–1^ blue light (BL) or darkness (DK) for 3 d. (C) The physical interaction of CNT2 and HBI1 analyzed in yeast cells. Yeast cells co-expressing CNT2/HBI1 were grown on basic (SD-W-L) or selective media with 5 mM 3AT (SD-W-L-H+3AT) in continuous 30 μmol m^–2^ s^–1^ blue light (BL) or darkness (DK) for 3 d at the top or cells co-expressing CCT2/HBI1 were grown on basic (SD-W-L) or selective media with 2 mM 3AT (SD-W-L-H+3AT) in darkness for 3 d at the bottom. (D) Schematic diagram of constructs expressing CNT2 tagged by the NLS sequence and CRY2 fused to YFP. (E) Co-localization assay showing that HBI1 co-localizes in the same NBs with CNT2 and CRY2 in tobacco cells. Dic, differential interference contrast. Scale bars=5μm.

It has been demonstrated that CRY1 regulates hypocotyl elongation under both low and high intensity of blue light, whereas CRY2 regulates this process mainly under low intensity of blue light (Lin *et al.*, 1998). Given the previous demonstration that the signaling mechanism of both CRY1 and CRY2 involves direct interactions with COP1, SPAs, and PIFs (Wang *et al.*, 2001; Yang *et al.*, 2001; Lian *et al.*, 2011; Liu *et al.*, 2011; Ma *et al.*, 2016; Pedmale *et al.*, 2016), we first examined whether CRY2 might also interact with HBI1 through yeast two-hybrid assay using the bait construct expressing the full-length CRY2 (Fig. 4A) and the prey construct expressing HBI1 (Fig. 2B). The results showed that the full-length CRY2 interacted with HBI1 in the dark and in blue light (Fig. 4B). To determine further whether the CRY2 N-terminus or C-terminus (designated as CNT2 and CCT2) might mediate the interaction of CRY2 with HBI1, we made bait constructs expressing CNT2–NLS and NLS–CCT2, respectively (Fig. 4A), and performed a yeast two-hybrid assay again with these bait constructs and the prey constructs expressing HBI1 and COP1. As shown in Fig. 4C, CNT2 interacted with HBI1 in blue light, but not in darkness, whereas CCT2 did not, but interacted with the positive control COP1 (Wang *et al.*, 2001). Moreover, we performed a protein co-localization study to confirm the interactions of CRY2 and CNT2 with HBI1 in tobacco cells using the constructs expressing CRY2–YFP and CNT2–NLS–YFP (Fig. 4D), and the construct expressing HBI1–CFP (Fig. 3A), respectively. The results showed that HBI1 was localized to the same NBs of CRY2 and CNT2 (Fig. 4E), indicating interactions of CRY2/CNT2 with HBI1. Taken together, these results suggest that, like CRY1, CRY2 interacts with HBI1 through its N-terminus.

### HBI1 positively regulates hypocotyl elongation under blue, red, and far-red light

**Fig. 5.**
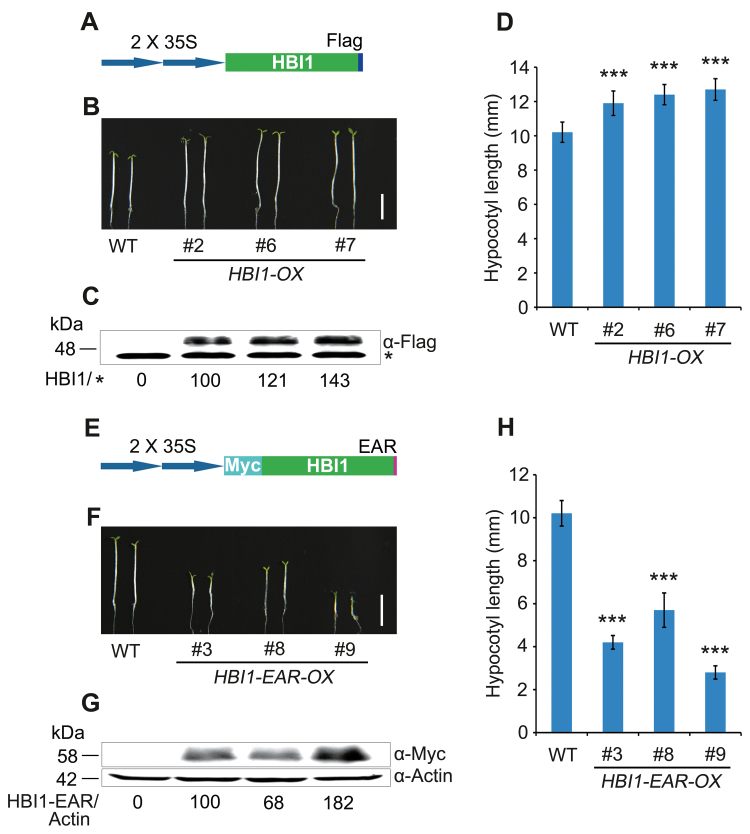
HBI1 acts as a positive regulator of hypocotyl elongation under blue light. (A and E) Schematic diagram depicting the constructs expressing fusion proteins of HBI1-Flag and Myc-HBI1–EAR used for transformation of WT plants. (B and F) Seedling phenotypes of *HBI1-OX* and *HBI1-EAR-OX* transgenic plants grown on half-strength MS medium under 2 μmol m^–2^ s^–1^ blue light for 5 d. Scale bars=5 mm. (C) Western blot analysis showing HBI1-Flag fusion protein expression in the corresponding lines shown in (B). An asterisk denotes a non-specific band recognized by the antibody, which served as a loading control. HBI1/* denotes the relative band intensities of HBI1-Flag normalized to the non-specific band and presented relative to that in *HBI1-OX* #2 set at unity. (D) Statistical analysis of hypocotyl length of seedlings shown in (B). Data are presented as means ±SD. Asterisks denote a signiﬁcant difference between the indicated lines and the WT (*t*-test, *P*<0.001), *n*=30. (G) Western blot analysis showing Myc-HBI1–EAR fusion protein expression in the corresponding lines shown in (F). Actin served as a loading control. HBI1-EAR/Actin indicates the relative band intensities of Myc-HBI1–EAR normalized to Actin and presented relative to that in *HBI1-EAR-OX* #3 set at unity. (H) Statistical analysis of hypocotyl length of seedlings shown in (F). Data are presented as means ±SD. Asterisks denote a signiﬁcant difference between the indicated lines and the WT (*t*-test, *P*<0.001), *n*=30.

It has been demonstrated that HBI1 functions in a triantagonistic cascade system downstream of BR, GA, temperature and light signaling pathways to promote hypocotyl elongation (Bai *et al.*, 2012*a*). Our demonstration that CRY1 physically interacts with HBI1 suggests that CRY1-mediated blue light inhibition of hypocotyl elongation might proceed, at least in part, through HBI1. To define a role for HBI1 in regulating hypocotyl elongation under blue light, we first generated transgenic lines overexpressing HBI1 tagged with Flag (*HBI1-OX*) (Fig. 5A, B), in which the expression of HBI1-Flag fusion protein was detected by western blot (Fig. 5C), and analyzed the hypocotyl elongation phenotype in blue light. The results showed that these lines exhibited a significantly taller hypocotyl phenotype than the WT under blue light (Fig. 5B, D). We then made a construct expressing Myc-HBI1 fused to the EAR motif (*HBI1-EAR*) (Fig. 5E), which is shown to inactivate the function of the native HBI1 and its close homologs (Bai *et al.*, 2012*a*; Liu *et al.*, 2013), and generated transgenic lines overexpressing Myc-HBI1–EAR (*HBI1-EAR-OX*). Analysis of the hypocotyl phenotype demonstrated that these lines had significantly shorter hypocotyls than the WT under blue light, and that the severity of this shortened hypocotyl phenotype was positively correlated with the HBI1–EAR protein levels (Fig. 5F–H), confirming a positive role for HBI1 in regulating hypocotyl elongation under blue light.

Next, we analyzed the blue light fluence rate response of *HBI1-OX* and *HBI1-EAR-OX* seedlings, and found that the hypocotyls of both lines were shortened as the fluence rate of blue light increased, and that the hypocotyls of *HBI1-OX* seedlings were always taller than those of the WT at all blue light fluence rate examined, whereas those of *HBI1-EAR-OX* seedlings were always shorter than those of the WT (Supplementary Fig. S1). However, the highest hypocotyl length difference between *HBI1-EAR-OX* and the WT was observed at a fluence rate <2 μmol m^–2^ s^–1^, indicating that HBI1 might function to promote hypocotyl elongation preferentially under low fluence rates of blue light. We further analyzed the hypocotyl phenotype of *HBI1-OX* and *HBI1-EAR-OX* plants in darkness, red, and far-red light. The results demonstrated that *HBI1-OX* seedlings developed slightly taller hypocotyls than the WT in these conditions, whereas *HBI1-EAR-OX* seedlings had dramatically shorter hypocotyls than the WT (Supplementary Fig. S2). Furthermore, we analyzed the red and far-red light fluence rate responses of *HBI1-OX* and *HBI1-EAR-OX* seedlings, and found that both lines basically displayed a similar tendency as observed under different fluence rates of blue light (Supplementary Fig. S3), and that the highest hypocotyl length difference between *HBI1-EAR-OX* and the WT under red and far-red light was observed at a fluence rate <30 μmol m^–2^ s^–1^and 1 μmol m^–2^ s^–1^, respectively (Supplementary Fig. S3). These results suggest that HBI1 might also promote hypocotyl elongation preferentially under low fluence rates of red and far-red light. Since the *HBI1-EAR-OX* lines that expressed high level of HBI1–EAR had extremely compact morphology and failed to survive after transfer to soil, we selected two heterozygous *HBI1-EAR-OX* lines (*HBI1-EAR-OX* #3 and #9) to analyze the hypocotyl phenotype in T_2_. As shown in Supplementary Fig. S4, the T_2_ siblings segregated from these heterozygous lines were divided into three different classes of seedlings with short, medium, and long hypocotyls at a ratio of ~1:2:1, further implying that the severity of the shortened hypocotyl phenotype is positively correlated with the HBI1–EAR protein levels. Taken together, these results suggest that HBI1 positively regulates hypocotyl elongation in darkness and in blue, red, and far-red light.

### HBI1 acts downstream of CRYs to promote hypocotyl elongation

**Fig. 6.**
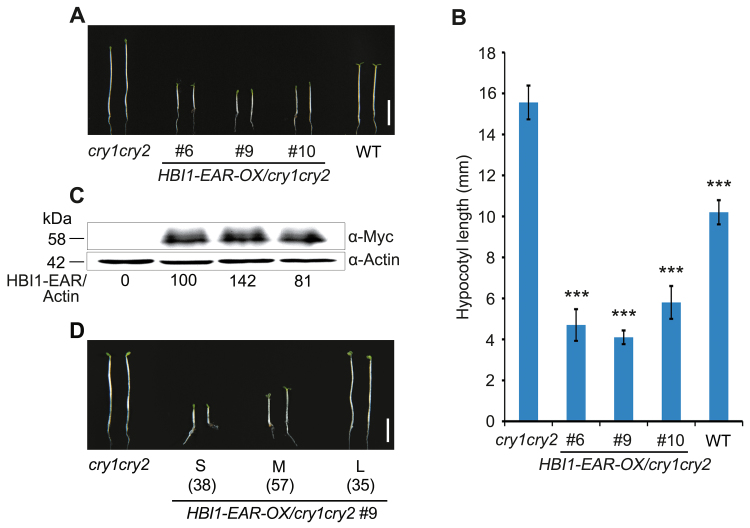
*HBI1* acts genetically downstream of *CRY* genes to promote hypocotyl elongation. (A) Seedling phenotypes of the transgenic lines overexpressing *HBI1-EAR* in the *cry1cry2* mutant background (*HBI1-EAR-OX/cry1cry2*) grown on half-strength MS medium under 2 μmol m^–2^ s^–1^ blue light for 5 d. Scale bar=5 mm. (B) Statistical analysis of hypocotyl length of seedlings shown in (A). Data are presented as means ±SD. Asterisks denote a signiﬁcant difference between the indicated lines and *cry1cry2* double mutant (*t*-test, *P*<0.001), *n*=30. (C) Western blot analysis showing Myc-HBI1–EAR fusion protein expression in corresponding lines shown in (A). Actin served as a loading control. HBI1-EAR/Actin indicates the relative band intensities of Myc-HBI1–EAR normalized to Actin and presented relative to that in *HBI1-EAR-OX/cry1cry2* #6 set at unity. (D) Seedling phenotype of siblings segregated from the T_2_ heterozygous *HBI1-EAR-OX/cry1cry2* #9 grown on half-strength MS medium under 2 μmol m^–2^ s^–1^ blue light for 5 d. Scale bar=5 mm. S, M, and L denote the segregated seedlings with short, medium, and long hypocotyls, respectively, and the numbers of these seedlings are shown under these letters.

With the demonstration that CRYs physically interact with HBI1, we next explored the genetic interaction of *CRY* genes with *HBI1* by generating transgenic *cry1cry2* mutant plants overexpressing Myc-HBI1–EAR (*HBI1-EAR-OX/cry1cry2*). As shown in Fig. 6A, B, *HBI1-EAR-OX/cry1cry2* lines developed dramatically shorter hypocotyls than the *cry1cry2* mutant under blue light. The expression of the Myc-HBI1–EAR fusion protein in these lines was detected by western blot (Fig. 6C). We selected a heterozygous *HBI1-EAR-OX/cry1cry2* line (*HBI1-EAR-OX/cry1cry2* #9) to analyze the hypocotyl phenotype in T_2_. As shown in Fig. 6D, the siblings segregated from the T_2_ heterozygous line were divided into three different classes of seedlings with short, medium, and very long hypocotyls at a ratio of ~1:2:1, further indicating that the severity of the shortened hypocotyl phenotype is positively correlated with the HBI1–EAR protein levels. These results suggest that *HBI1* acts downstream of *CRY* genes to regulate hypocotyl elongation under blue light.

We also examined the phenotypes of adult plants of the WT, *cry1cry2*, *HBI1-OX*, *HBI1-EAR-OX*, and *HBI1-EAR-OX/cry1cry2*, and observed that *HBI1-OX* and *cry1cry2* mutant plants developed longer rosette leaves and siliques than the WT, whereas *CRY1-OX* plants had shorter rosette leaves and siliques than the WT, and that *HBI1-EAR-OX* plants hardly developed functional rosette leaves and siliques (Supplementary Fig. S5). Moreover, *HBI1-EAR-OX/cry1cry2* plants had much shorter rosette leaves and siliques than the *cry1cry2* mutant. Taken together, these results suggest that CRYs and HBI1 act antagonistically to regulate not only hypocotyl elongation during early seedling photomorphogenesis, but also leaf and silique elongation during vegetative and reproductive development.

### CRYs and HBI1 antagonistically regulate the expression of a set of genes related to cell elongation

**Fig. 7.**
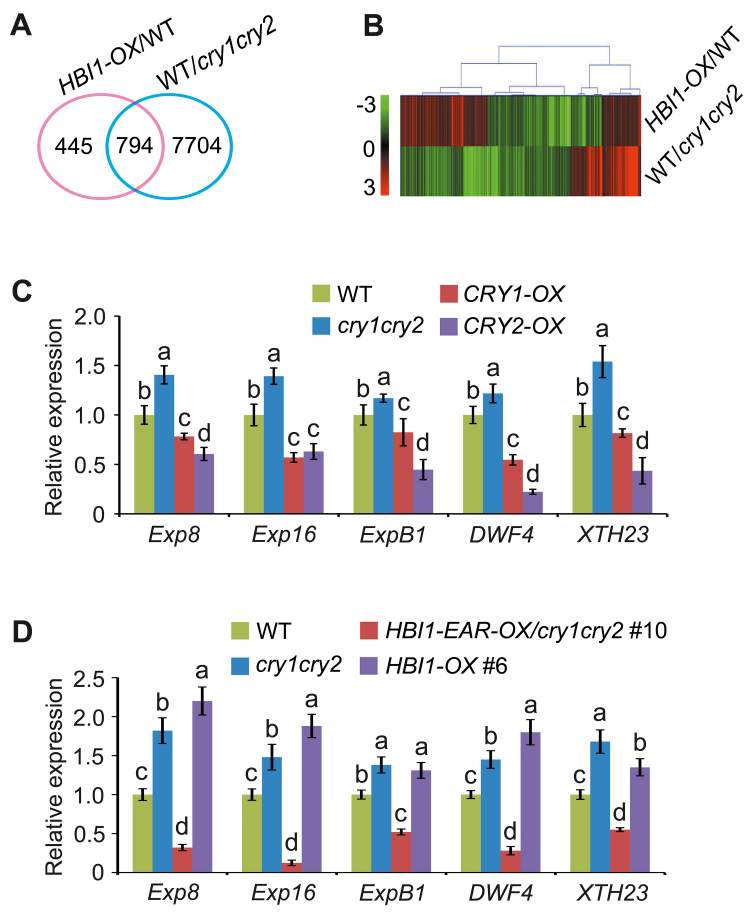
CRYs and HBI1 antagonistically regulate the expression of genes promoting cell elongation. (A) Venn diagram showing the overlap of genes regulated by HBI1 and CRYs. (B) Hierarchical cluster analysis of 794 overlapping genes shown in (A). Red and green colors in the heatmap represent induced and repressed genes, respectively. The gradient bar denotes the log_2_-fold change relative to the control sample. (C and D) qRT-PCR analyses showing the expression of direct target genes of HBI1 in multiple genotypes indicated. All the seedlings were grown in darkness for 5 d and then transferred to 30 μmol m^–2^ s^–1^ blue light for 0.5 h. Expression levels were normalized to an internal control *PP2A*, and the WT level was arbitrarily set to 1. Data are represented as means ±SD (*n*=3). The lower case letters ‘a’ to ‘d’ indicate statistically signiﬁcant differences among means for gene expression levels of the indicated genotypes, as determined by Tukey’s LSD test (*P*≤0.01).

To explore whether the regulation of hypocotyl elongation by CRYs and HBI1 might proceed through the co-regulation of genes related to cell elongation, we first compared the 8498 genes regulated by CRYs with the 1239 genes regulated by HBI1, which were obtained through RNA-Seq (Fan *et al.*, 2014; He *et al.*, 2015). We found that, of the 1239 HBI1-controlled genes, 794 genes (64.1%) were also affected by CRYs (Fig. 7A). A heatmap revealed that, among these co-regulated genes, 393 genes (50%) were affected in the opposite way (Fig. 7B). GO analysis suggests that the genes oppositely regulated by CRYs and HBI1 were largely involved in the regulation of the biological processes including cell wall organization and cell wall loosening, whereas those co-regulated by CRYs and HBI1 in the same direction were not (Supplementary Fig. S6).

We performed qRT-PCR analysis of five representative direct target genes of HBI1, which are positively regulated by HBI1 and known to promote hypocotyl elongation (Fan *et al.*, 2014), in WT, *cry1cry2* mutant, *CRY1-OX*, and *CRY2-OX* seedlings that were exposed to blue light. As shown in Fig. 7C, the expression of these genes was enhanced to varying degrees in the *cry1cry2* mutant, but reduced to varying degrees in *CRY1-OX* and *CRY2-OX* plants. We also analyzed the expression of these genes in *HBI1-EAR-OX/cry1cry2* and *HBI1-OX* seedlings exposed to blue light and found that these genes were expressed at significantly lower levels in *HBI1-EAR-OX/cry1cry2* than in the *cry1cry2* mutant and *HBI1-OX* seedlings (Fig. 7D). Taken together, these results indicate that CRY inhibition of hypocotyl elongation is mediated, at least in part, through repression of the expression of HBI1 target genes that act to promote cell elongation.

### CRY1 inhibits the transcriptional activity of HBI1 through its N-terminus

**Fig. 8.**
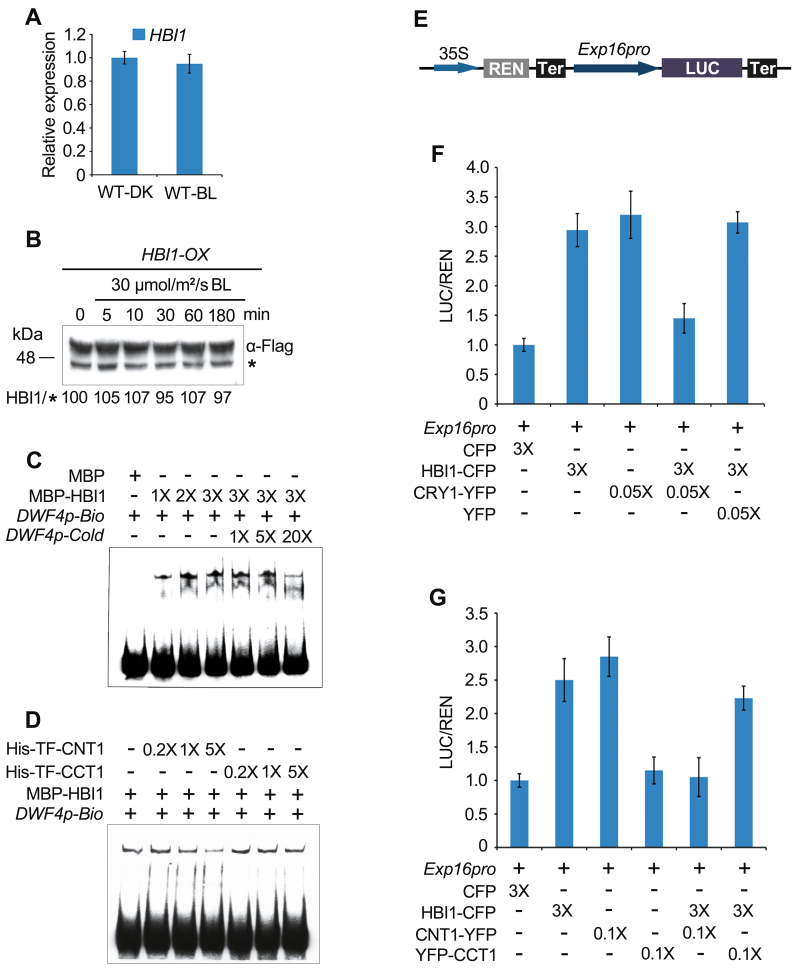
CRY1 represses HBI1’s transcriptional activity through CNT1 by inhibiting the DNA binding activity of HBI1. (A) qRT-PCR analysis showing the expression of *HBI1* in WT seedlings grown in darkness for 5 d with adaption in darkness (WT-DK) or an exposure to 30 μmol m^–2^ s^–1^ blue light (WT-BL) for 0.5 h. Expression levels were normalized to an internal control *PP2A*, and the WT-DK level was arbitrarily set to 1. Data are represented as means ±SD (*n*=3). (B) Immunoblot analysis of the HBI1-Flag fusion protein level in *HBI1-OX* seedlings grown in darkness for 5 d and then exposed to 30 μmol m^–2^ s^–1^ blue light for the durations indicated. An asterisk denotes a non-specific band serving as a loading control. HBI1/* indicates the relative band intensities of HBI1-Flag normalized to the non-specific band and presented relative to that in darkness set at unity. (C and D) EMSAs showing CNT1 inhibition of HBI1 DNA binding ability. EMSA was performed with MBP–HBI1 and MBP proteins using a labeled probe composed of a DNA fragment of the promoter of *DWF4* and biotin (*DWF4p-Bio*). 1 × 2×, 3×, and 1×, 5×, 20× in (C) indicate the amounts of MBP–HBI1 and cold competitor DNA (*DWF4p-Cold*) relative to the initial concentration of MBP–HBI1 and *DWF4p-Bio* probes, respectively. 0.2×, 1×, and 5× in (D) indicate the amounts of His-TF–CNT1 and His-TF–CCT1 relative to one-tenth the amount of MBP–HBI1. Signals at the bottom indicate free probes. (E) Schematic diagram of the construct of the Dual-LUC assay reporter expressing REN under the control of 35S promoter and LUC under the control of the *Exp16* promoter (*Exp16*_*pro*_). (F and G) Dual-LUC assays showing that CRY1 represses the transcriptional activity of HBI1 through CNT1. Tobacco leaves were transfected with *Agrobacterium* in different combinations of effectors and the *Exp16*_*pro*_ reporter, and then kept in dim light for 3 d. 0.05×, 3× in (F) and 0.1×, 3× in (G) indicate the culture volume of effectors relative to that of the *Exp16*_*pro*_ reporter. The ratio of LUC activity relative to REN activity of the expression control (CFP as effector) was arbitrarily set to 1, to which the ratios of other groups were normalized. Error bars represent ±SD (*n*=4).

To explore how CRY1 might regulate HBI1 activity, we analyzed whether *HBI1* gene expression or HBI1 protein expression might be regulated by blue light using the dark-grown WT seedlings or *HBI1-OX* seedlings exposed to blue light for increasing lengths of time. The results showed that neither *HBI1* gene expression nor HBI1 protein expression was affected by blue light (Fig. 8A, B). We then explored whether CRY1 might regulate the DNA binding activity of HBI1 through CRY1–HBI1 interaction. To do this, we performed an EMSA to investigate whether the capacity of HBI1 to bind to the sequence of the promoter of one HBI1 target gene, *DWF4* (*DWARF 4*), whose expression is inhibited by CRYs (Fig. 7C), might be inhibited by CNT1. The control experiment showed that HBI1 bound to the promoter sequence of the *DWF4* gene that contains the E box sequence (CANNTG) in a protein concentration-dependent manner, and that the binding capacity was reduced by the competition from a cold competitor (Fig. 8C), whereas this binding was not detectable with the control protein MBP. As anticipated, the DNA fragment bands shifted by HBI1 decreased as the amounts of CNT1 increased (Fig. 8D), whereas they were not affected by increasing amounts of CCT1. These results indicated that CNT1 but not CCT1 inhibited the DNA binding activity of HBI1 *in vitro*.

Next, we performed Dual-LUC assays to examine CRY1 regulation of HBI1 transcriptional activity in tobacco cells. To do this, we isolated the 2 kb promoter region of the *Exp16* gene (*Exp16*_*pro*_), which is a HBI1 direct target gene (Fan *et al.* 2014), and made the reporter construct expressing *LUC* under the control of *Exp16*_*pro*_ (Fig. 8E). The effector constructs were transiently co-expressed in tobacco leaf cells with the reporter construct in different combinations. The results showed that, consistent with HBI1 being a positive regulator of *Exp16* (Fan *et al.*, 2014), HBI1 alone strongly stimulated the *Exp16*_*pro*_:*LUC* reporter activity (Fig. 8F). However, when CRY1 was co-expressed with HBI1, the activity of the *Exp16* promoter was dramatically repressed (Fig. 8F). We then analyzed the effects of CNT1 and CCT1 on HBI1 regulation of the *Exp16*_*pro*_:*LUC* reporter activity. As shown in Fig. 8G, HBI1 induction of *Exp16*_*pro*_:*LUC* activity was considerably inhibited by CNT1, but barely affected by CCT1. In addition, HBI1 activity was inhibited by CRY1 and CNT1 in a dose-dependent manner (Supplementary Fig. S7). Taken together, these results suggest that CNT1-mediated interaction of CRY1 with HBI1 leads to inhibition of HBI1 DNA binding activity and its transcriptional activity.

## Discussion

Previous reports have revealed that CRY1 and CRY2 interact with COP1 through their C-terminus to inhibit COP1 activity and enhance the accumulation of HY5 to promote photomorphogenesis (Yang *et al.*, 2000, 2001; Wang *et al.*, 2001). Moreover, later studies introduced SPAs into CRY signaling pathway and demonstrated that CRYs interact with SPAs in a blue light-dependent manner, resulting in inhibition of the function of the COP1–SPA complex and further repression of COP1 activity (Lian *et al.*, 2011; Liu *et al.*, 2011; Zuo *et al.*, 2011). These studies led to the establishment of the CRY signaling cascade, which consists of CRYs, SPAs, COP1, and HY5. However, recent studies suggest that the CRY1 N-terminus and C-terminus probably regulate hypocotyl elongation through different mechanisms (W.X. Wang *et al.*, 2016) and that the CRY1 N-terminus is able independently to confer enhanced blue light inhibition of hypocotyl elongation (He *et al.*, 2015). Therefore, the CRY1 N-terminus may function independently of the CRY1 C-terminus to mediate CRY1 signaling by interacting with downstream components. Most recently, two reports have shown that CRYs interact with PIF bHLH transcription factors to regulate their transcriptional activity and mediate responses to canopy shade, low blue light, or high temperature (Ma *et al.*, 2016; Pedmale *et al.*, 2016).

**Fig. 9.**
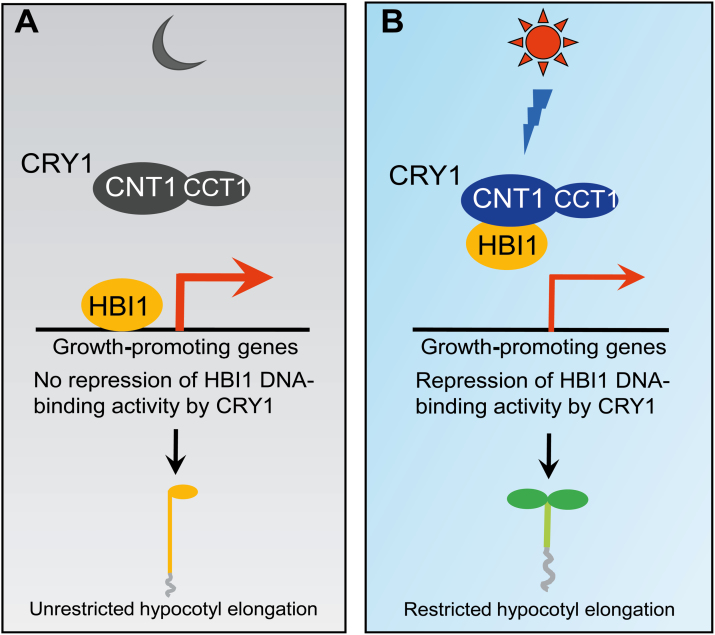
A model describing regulation of HBI1 transcription by CRY1. (A) In darkness, CRY1 is inactive and HBI1 is able to bind to the promoters of its direct target genes promoting cell elongation and activating their expression, leading to enhanced hypocotyl elongation. (B) Upon blue light irradiation, CRY1 is activated and able to interact with HBI1 through its N-terminus (CNT1) to inhibit HBI1 binding to its target genes, resulting in repression of expression of these genes and inhibited hypocotyl elongation. CCT1 denotes the CRY1 C-terminus. Thicker and thinner red arrows denote higher and lower gene expression, respectively.

CRY1 is the primary blue light photoreceptor mediating blue light inhibition of hypocotyl elongation. The loss-of-function mutant of Arabidopsis CRY1 shows a dramatically enhanced hypocotyl elongation phenotype, whereas transgenic plants overexpressing CRY1 display a considerably shortened hypocotyl phenotype (Ahmad and Cashmore, 1993; Lin *et al.*, 1996). In this study, we further defined a role for a bHLH transcription factor HBI1 in promoting hypocotyl growth in blue, red, and far-red light. The results presented here demonstrate that Arabidopsis CRY1 interacts with HBI1 to inhibit its DNA binding activity and hypocotyl elongation. Specifically, we demonstrate by combined approaches of yeast two-hybrid assay, pull-down assay, protein co-localization, and co-IP that CRY1 interacts with HBI1 through its N-terminus in a blue-light-dependent manner. The significance of the CRY1–HBI1 interaction is attested to by the following demonstrations. (i) HBI1 acts downstream of CRYs to regulate hypocotyl elongation under blue light. (ii) CRYs act to co-regulate a set of genes that are involved in regulating cell elongation antagonistically with HBI1. (iii) The CRY1 N-terminus inhibits HBI1–DNA binding activity in vitro. (iv) Both CRY1 and its N-terminus are able to inhibit HBI1 transcriptional activity in tobacco leaf cells. Based on our results, we propose a model describing CRY1 regulation of hypocotyl elongation via its interaction with HBI1 (Fig. 9). In darkness, CRY1 is not active and cannot interact with HBI1 protein. Hence, HBI1 is fully active, and able to bind to its target genes and regulate their expression, leading to active hypocotyl elongation. Upon blue light irradiation, CRY1 is activated, and able to interact with HBI1 to inhibit its binding to target genes, leading to inhibition of hypocotyl elongation.

It should be noted that we observed that CNT1 interacted with HBI1 regardless of blue light in yeast two-hybrid assays, whereas CNT2 interacted with HBI1 in blue light, but not in darkness (Figs 2C, 4C). These differences might result from the amino acid sequence dissimilarity between CNT1 and CNT2. Although CRY1 and CRY2 are homologous proteins, they have different functions and protein properties. For example, CRY1 plays a role in inhibiting hypocotyl elongation under low, middle, and high fluence rates of blue light, whereas CRY2 performs this role primarily under a low intensity of blue light (Lin *et al.*, 1998). Consistent with this, CRY2 is degraded under a high intensity of blue light, whereas CRY1 is stable (Shalitin *et al.*, 2002). The amino acid sequence differences between CRY1 and CRY2 might also lead to these differences. In our Dual-LUC assays, we found that, similar to a previous report (Ma *et al.*, 2016), CRY1 or CNT1 alone is somehow able to activate the *Exp16*_*pro*_ reporter in tobacco cells (Fig. 8F, G). Given the demonstrations that CRY1 is not a transcription factor and that soybean CRY2a interacts with CIB1 in a blue-light-dependent manner to inhibit CIB1 DNA binding activity and repress leaf senescence (Meng *et al.*, 2013), we postulate that CRY1 activation of *Exp16*_*pro*_ activity is not likely to be mediated through a direct mechanism, namely CRY1 binding to *Exp16*_*pro*_. It is likely that CRY1–HBI1 interaction might lead to a decrease in HBI1–DNA binding and transcriptional activity.

In view of the fact that the signaling mechanism of both CRY1 and CRY2 involves physical interactions with COP1, SPAs, and PIFs, and our demonstration that CRY2 also interacts with HBI1 in yeast cells and tobacco cells, it is possible that the signaling mechanism of CRY2 also involves interaction with HBI1 under a low intensity of blue light. Moreover, our observation that HBI1 acts to promote hypocotyl elongation in red and far-red light (Supplementary Figs S2, S3) suggests a possible role for HBI1 in mediating phytochrome signaling. Given previous demonstrations that phytochromes interact with the cryptochrome-interacting proteins such as COP1 and SPAs (Seo *et al.*, 2004; Jang *et al.*, 2010; Lu *et al.*, 2015; Sheerin *et al.*, 2015), and that cryptochromes interact with the phytochrome-interacting factor PIF4 to mediate light signaling (Ma *et al.*, 2016; Pedmale *et al.*, 2016), it is possible that phytochromes may interact directly with HBI1 to regulate hypocotyl elongation. On the other hand, phytochromes might influence HBI1 function indirectly by regulating the biosynthesis of the endogenous phytohormones BR and GA based on the following demonstrations: (i) PIF4 and PIF5 bind to the promoter regions of the key BR biosynthetic genes *DWF4* and *BR6ox2* to promote their expression directly (Wei *et al.* 2017); (ii) phytochromes regulate GA biosynthesis through PIF3-LIKE5 (PIL5), which represses the expression of GA biosynthetic genes *GA3ox1* and *GA3ox2* and activates the expression of a GA catabolic gene *GA2ox* in Arabidopsis (Oh *et al.* 2006); and (iii) HBI1 is involved in the signaling pathway of BR and GA (Bai *et al.*, 2012*a*).

The bHLH-type proteins constitute a large family of transcription factors in eukaryotes, which is specifically classified into dozens of subfamilies with a total of ~170 members in Arabidopsis (Carretero-Paulet *et al.*, 2010). A previous study reported that bHLH factors are capable of forming homodimers or heterodimers through the HLH domains and binding to speciﬁc DNA sequences through the basic domains (Toledo-Ortiz *et al.*, 2003). HBI1 was identified as a typical DNA-binding bHLH protein and closely related to many bHLH factors belonging to different subfamilies. Among them, CIBs (CIB4/5), CILs (CIL1/2), and BEEs (BEE1/2/3) are also shown to regulate hypocotyl elongation (Friedrichsen *et al.*, 2002; Ikeda *et al.*, 2012). Based on our results showing that CRY1 may interact with these bHLH proteins through its N-terminus, we postulate that these interactions may also result in inhibition of their DNA binding activity and reduced expression of their target genes that are involved in positively regulating cell elongation.

 It has been demonstrated that the regulatory module PRE1–IBH1–HBI1, in which PRE1 interacts with IBH1 and suppresses the inhibitory effect of IBH1 on HBI1 to promote HBI1 activity, plays an important role in the control of cell elongation downstream of a broad range of signals (Bai *et al.*, 2012*a*), such as BR, GA, light, and temperature. Furthermore, the BZR1–PIF4–DELLA module, which also integrates BR and GA signaling (Bai *et al.*, 2012*b*; Ikeda *et al.*, 2012; Oh *et al.*, 2012), is involved in the regulation of HBI1 activity by directly promoting *PRE* expression via the BZR1–PIF4 complex and repressing *IBH1* expression via the BZR1 factor. Therefore, it appears that the PRE–IBH1–HBI1 module functions downstream of the BZR1–PIF4–DELLA module to respond to different signals to regulate hypocotyl elongation. Recently, several studies have shown the functional connections between CRYs and PIFs in the regulation of plant responses to temperature and shade (Ma *et al.*, 2016; Pedmale *et al.*, 2016). The present study reveals the physical interaction of CRY1 with HBI1, which further demonstrates an important role for HBI1 in the integration of light and phytohormone signaling.

## Supplementary data

Supplementary data are available at *JXB* online.

Table S1. Constructs and primers used in this study.

Fig. S1. HBI1 promotes hypocotyl elongation under different fluence rates of blue light.

Fig. S2. HBI1 positively regulates hypocotyl elongation under different light conditions.

Fig. S3. HBI1 promotes hypocotyl elongation under different fluence rates of red and far-red light.

Fig. S4. Phenotype analyses of siblings segregated from T_2_ heterozygous lines of *HBI1-EAR-OX* #3 and #9.

Fig. S5. Phenotypic analyses of adult plants of the WT, *HBI1-OX* #6, *cry1cry2*, *HBI1-EAR-OX* #3, *HBI1-EAR-OX/cry1cry2* #6, and *CRY1-OX*.

Fig. S6. Gene Ontology (GO) analysis of overlapping genes regulated by HBI1 and CRYs.

Fig. S7. Dual-LUC assays showing CRY1 inhibition of the transcriptional activity of HBI1 through CNT1 in a dose-dependent manner.

Supplementary Material

## Abbreviations

3AT: 3-amino-1,2,4-triazole
bHLH: basic helix–loop–helix
BR: brassinosteroid
BEE2: BR enhanced expression 2
CCT1: cryptochrome 1 C-terminal domain
CCT2: cryptochrome 2 C-terminal domain
CIB1: cryptochrome interacting basic helix–loop–helix 1
CIL1: CIB1-like protein 1
CNT1: cryptochrome 1 N-terminal domain
CNT2: cryptochrome 2 N-terminal domain
CO: CONSTANS
COP1: constitutively photomorphogenic 1
CRY1: cryptochrome 1
CRY2: cryptochrome 2
DWF4: dwarf 4
GA: gibberellin
HBI1: homolog of BEE2 interacting with IBH 1
IBH1: ILI1-binding bHLH protein 1
NB: nuclear body
PRE1: paclobtrazol resistance 1
