# Supplementary material for: CRY1 interacts directly with HBI1 to regulate its transcriptional activity and photomorphogenesis in Arabidopsis
Source: J Exp Bot. 2018 Jun 1;69(16):3867–81. doi: 10.1093/jxb/ery209 (PMC6054188; doi:10.1093/jxb/ery209)
Supplement: Supplementary Material [file ery209_suppl_supplementary_table_figures.pdf]

Supplementary Data

Table S1. Constructs and primers used in this study.

Assays	Constructs	Template genes	Primers	Primer sequences
Yeast two-hybrid	*pGBKT7-CNT1-NLS*	*CRY1*	*CNT1-F-EcoR Ⅰ*	5’>CG*GAATTC*ATGTCTGGTTCTGTATCTGGTT<3’
			CNT1-NLS-R-Sal Ⅰ	5’>GGG*GTCGAC*TCATGGATCTTCCACCTTTCTCTTCTTCTTTGGTGAAAGCGCTTCATGAAGCCGT<3’
	pGBKT7-NLS-CCT1	*CRY1*	*NLS-CCT1-F-EcoR Ⅰ*	5’>CG*GAATTC*ATGCCAAAGAAGAAGAGAAAGGTGGAAGATCCACAGATGTGGCAACTAGAAGCTG<3’
			CCT1-R-Sal Ⅰ	5’>GGG*GTCGAC*TTACCCGGTTTGTGAAAGCCGT<3’
	pGBKT7-CNT2-NLS	*CRY2*	*CNT2-F-Mfe Ⅰ*	5’>GGC*CAATTG*ATGAAGATGGACAAAAAGACTA<3’
			CNT2-NLS-R-Sal Ⅰ	5’>GGG*GTCGAC*TCATGGATCTTCCACCTTTCTCTTCTTCTTTGGAATAGCTTTAGCTAGTAGCTCA<3’
	pGBKT7-NLS-CCT2	*CRY2*	*NLS-CCT2-F-Mfe Ⅰ*	5’>GGC*CAATTG*ATGCCAAAGAAGAAGAGAAAGGTGGAAGATCCATCAAGAACCCGTGAAGCACAGA<3’
			CCT2-R-Sal Ⅰ	5’>GGG*GTCGAC*TCATTTGCAACCATTTTTTCCC<3’
	pGBKT7-CRY2	*CRY2*	*CNT2-F-Mfe Ⅰ*	As above
			CCT2-R-Sal Ⅰ	As above
	pGADT7-HBI1	*HBI1*	*HBI1-F-EcoR Ⅰ*	5’>CG*GAATTC*ATGTTGGAAGGTCTTGTCTCT<3’
			HBI1-R-X	5’>CCC*CTCGAG*TTAGTAATGAAAA

			ho Ⅰ	CCGAGGCTA<3’
	pGADT7-CIB1	*CIB1*	*CIB1-F-EcoR Ⅰ*	5’>CG*GAATTC*ATGAATGGAGCTATAGGAGGTG<3’
			CIB1-R-Xho Ⅰ	5’>CCC*CTCGAG*TCAAACTCCTAAATTGCCATAG<3’
Colocal-ization	*pHB-CNT2-NLS-YFP*	*CRY2*	*CNT2-F-Bcl Ⅰ*	5’>GGC*TGATCA*ATGAAGATGGACAAAAAGACTA<3’
			CNT2-NLS-R-Spe Ⅰ	5’>GG*ACTAGT*TCATGGATCTTCCACCTTTCTCTTCTTCTTTGGAATAGCTTTAGCTAGTAGCTCA<3’
	pHB-HBI1-CFP	*HBI1*	*HBI1-F-BamH Ⅰ*	5’>GCC*GGATCC*ATGTTGGAAGGTCTTGTCTCT<3’
			HBI1-R-Spe Ⅰ	5’>GG*ACTAGT*GTAATGAAAACCGAGGCTAGATG<3’
	pHB-CFP-CIB1	*CIB1*	*CIB1-F-BamH Ⅰ*	5’>GCC*GGATCC*ATGAATGGAGCTATAGGAGGTG<3’
			CIB1-R-Spe Ⅰ	5’>GG*ACTAGT*TCAAACTCCTAAATTGCCATAG<3’
	pHB-CIL1-CFP	*CIL1*	*CIL1-F-BamH Ⅰ*	5’>GCC*GGATCC*ATGGATTTAAGTGCGAAAGATG<3’
			CIL1-R-Spe Ⅰ	5’>GG*ACTAGT*TGGCTCAACCTTCATATTTGCA<3’
	pHB-CFP-BEE2	*BEE2*	*BEE2-F–BamH I*	5’>GCC*GGATCC*ATGGACTTGTCTGTACTTGATA<3’
			BEE2-R–Spe I	5’>GG*ACTAGT*TTACTTGAGGCTGAAGAAATTG<3’
	pHB-CO-CFP	*CO*	*CO-F–BamH I*	5’>GCC*GGATCC*ATGTTGAAACAAGAGAGTAACG<3’
			CO-R–Spe I	5’>GG*ACTAGT*GAATGAAGGAACAATCCCATAT<3’

Pull-down	*pCold-TF-CNT1*	*CRY1*	*CNT1-F-EcoR Ⅰ*	5’>CG*GAATTC*ATGTCTGGTTCTGTATCTGGTT<3’
			CNT1-R-Sal Ⅰ	5’>GGG*GTCGAC*TCATGAAAGCGCTTCATGAAGCCGT<3’
	pCold-TF-CCT1	*CRY1*	*CCT1-F-EcoR Ⅰ*	5’>CG*GAATTC*ATGCAGATGTGGCAACTAGAAGCTG<3’
			CCT1-R-Sal Ⅰ	5’>GGG*GTCGAC*TTACCCGGTTTGTGAAAGCCGT<3’
	pMAL-c2X-HBI1	*HBI1*	*HBI1-F-EcoR Ⅰ*	5’>CG*GAATTC*ATGTTGGAAGGTCTTGTCTCT<3’
			HBI1-R-Xho Ⅰ	5’>CCC*CTCGAG*TTAGTAATGAAAACCGAGGCTA<3’
Co-IP	*pHB-YFP-NLS-GUS-CCT1*	*GUS-CCT1*	*NLS-GUS-F-Bgl II*	5’>GA*AGATCT*ATGCCAAAGAAGAAGAGAAAGGTGGAAGATCCAATGTTACGTCCTGTAGAAACCC<3’
			CCT1-R-Spe Ⅰ	5’>GG*ACTAGT*TTACCCGGTTTGTGAAAGCCGT<3’
	pHB-HBI1-Flag	*HBI1*	*HBI1-F-BamH Ⅰ*	5’>GCC*GGATCC*ATGTTGGAAGGTCTTGTCTCT<3’
			HBI1-R-Spe Ⅰ	5’>GG*ACTAGT*GTAATGAAAACCGAGGCTAGATG<3’
EMSA			*DWF4p-F*	5’>ACGGTGGATGAAAGTTATAGTACTATTAGCCAGAGACAATTGATTATAGATATATCCATTAATCCATGATATTTATGATA<3’
			DWF4p-R	5’>TATCATAAATATCATGGATTAATGGATATATCTATAATCAATTGTCTCTGGCTAATAGTACTATAACTTTCATCCACCGT<3’
			DWF4p-R-Biotin	5’>TATCATAAATATCATGGATTAATGGATATATCTATAATCAATTGTCTCTGGCTAATAGTACTATAACTTTCATCCACCGT<3’

Dual-LUC	*pGreen0800-Exp16pro*	*Exp16pro*	*Exp16pro-F-Xho* Ⅰ<br><br>*Exp16pro-R-Spe* Ⅰ	5’>CCC*CTCGAG*GATTAGATAACAAACTTTACTT<3’<br><br>5’>GG*ACTAGT*TTTGGTAAGTATTTTTAATTGA<3’
Transform-ation	*pHB-HBI1-Flag*<br><br>*pHB-Myc-HBI1-EAR*	*As above*<br><br>*HBI1*	*As above*<br><br>*HBI1-F-BamH* Ⅰ<br><br>*HBI1-EAR-R-Spe* Ⅰ	As above<br><br>As above<br><br>5’>GG*ACTAGT*TTAAGCGAAACCCAAACGGAGTTCTAGATCCAGATCGAGAAGCTTGTAATGAAAACCGAGGCTAGA<3’
Quantita-tive RT-PCR		*PP2A*	*PP2A RT-F*	5’>TATCGGATGACGATTCTTCGTGCAG<3’
			PP2A RT-R	5’>GCTTGGTCGACTATCGGAATGAGAG<3’
		Exp8	*Exp8 RT-F*	5’>TCCTCCTCTTCAGCATTTCGACCT<3’
			Exp8 RT-R	5’>CTTGCCACGACTGTGTTTTTGAGC<3’
		Exp16	*Exp16 RT-F*	5’>CGCTCTCATTTCGACCTCGCCAT<3’
			Exp16 RT-R	5’>CGTTCGTAATCAGCACCAAGTT<3’
		ExpB1	*ExpB1 RT-F*	5’>AACTGATTATTGGTTATCGCTT<3’
			ExpB1 RT-R	5’>CTTGAGTGGTCCTTCGACGATG<3’
		DWF4	*DWF4 RT-F*	5’>CATAAAGCTCTTCAGTCACGA<3’

			DWF4 RT-R	5’>CGTCTGTTCTTTGTTTCCTAA<3’
		XTH23	*XTH23 RT-F*	5’>GCTGGAACTGTCACCGCTTAC<3’
			XTH23 RT-R	5’>TCAAAGTCAATCTCGTCCCATGT<3’
		HBI1	*HBI1 RT-F*	5’>TGCCTGGATGCAATAAGGTCACAG<3’
			HBI1 RT-R	5’>TGGAGCTTCGATAGATGTCGTTTGG<3’

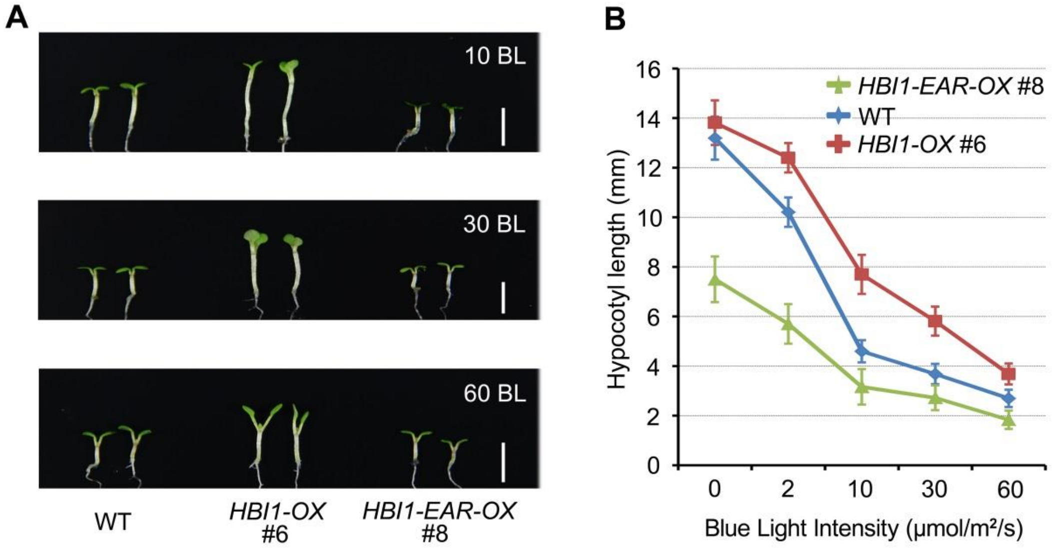

Supplementary Fig. S1. HBI1 promotes hypocotyl elongation under different fluence rates of blue light.

(A) Seedling phenotypes of WT and transgenic lines indicated grown on half-strength MS medium under different fluence rates of blue light for 5 days. Bars=5 mm. 10 BL, 30 BL, and 60 BL denote 10, 30, and 60 μmol/m^2/s blue light, respectively. (B) Quantitative analyses of hypocotyl length of seedlings of indicated lines in different blue light fluence rate indicated. Data are presented as means ± SD, n = 30.

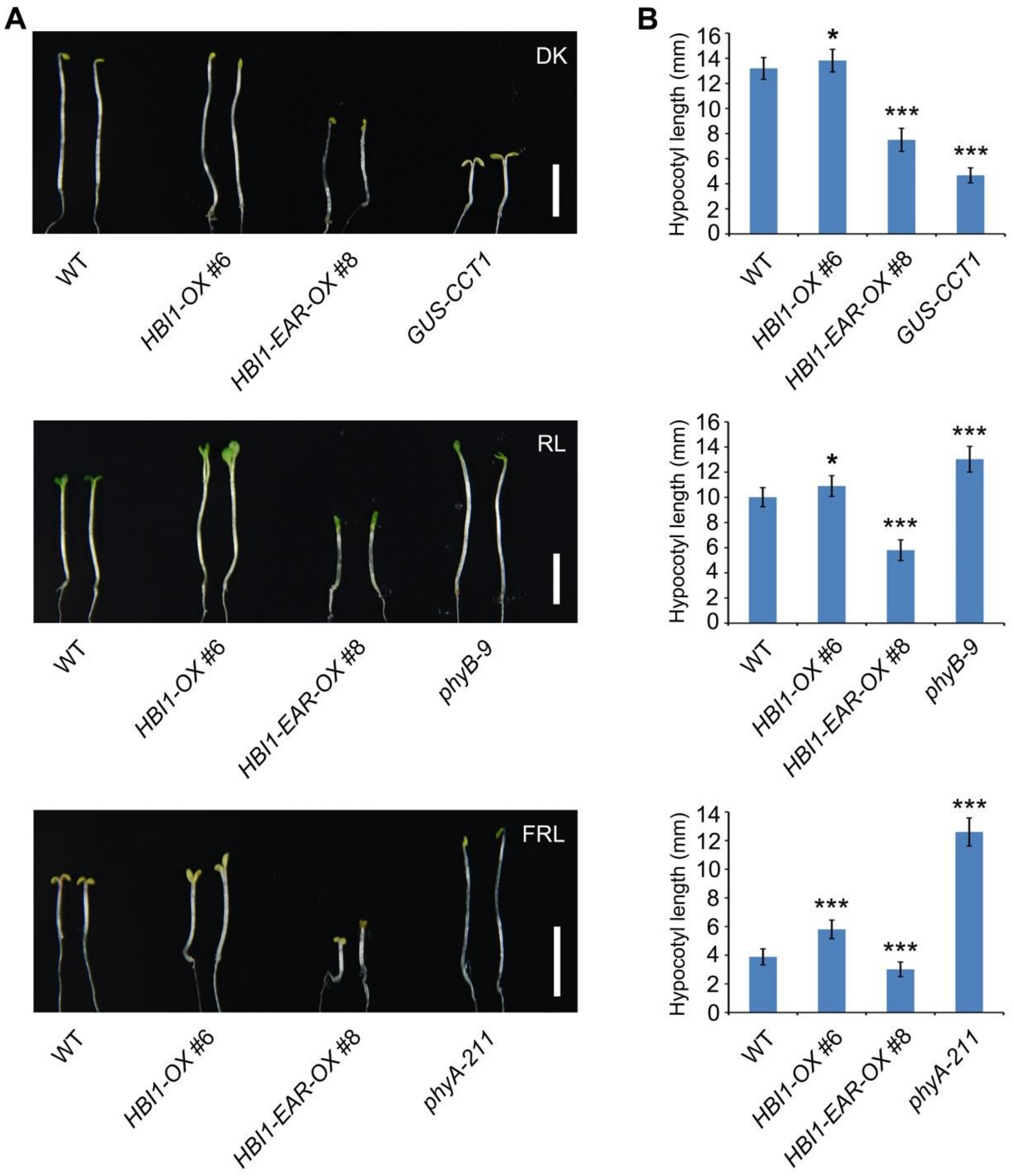

Supplementary Fig. S2. HBI1 positively regulates hypocotyl elongation under different light conditions.

(A) Seedling phenotypes of WT plants and transgenic lines indicated grown on half-strength MS medium under different light conditions for 5 days. Bars=5 mm. DK, darkness; RL, 30 μmol/m^2/s red light; FRL, 1 μmol/m^2/s far-red light. Transgenic *GUS-CCT1* line, and *phyB-9* and *phyA-211* mutants served as controls in DK, RL, and FRL, respectively. (B) Corresponding quantitative analyses of hypocotyl length of the seedlings shown in (A). Data are presented as means ± SD. Single asterisk denotes significant difference between the indicated line and WT in the same group (t test,

P<0.05) and asterisks denote significant difference between the indicated line and WT in the same group (t test, P<0.001), n = 30.

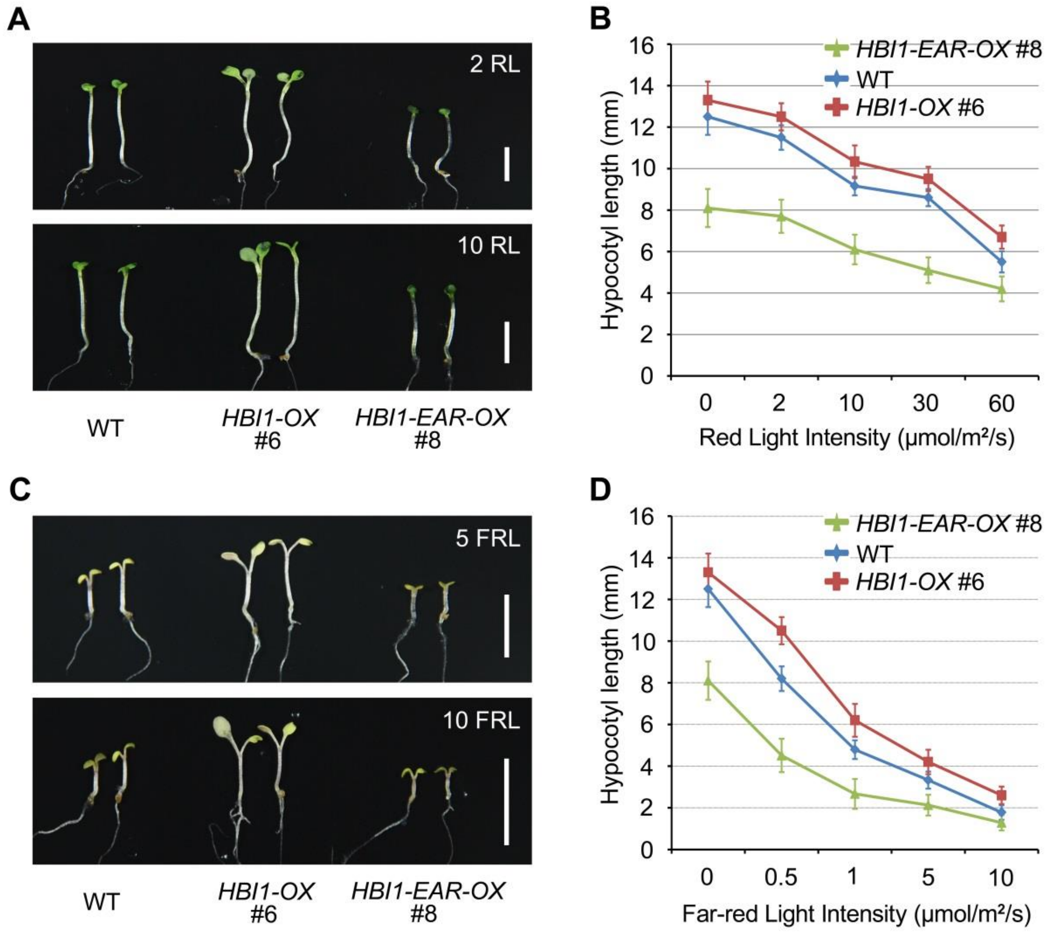

Supplementary Fig. S3. HBI1 promotes hypocotyl elongation under different fluence rates of red and far-red light.

(A and C) Seedling phenotypes of WT and transgenic lines indicated grown on half-strength MS medium under different fluence rates of red and far-red light for 5 days, respectively. 2 RL and 10 RL denote 2 and 10 μmol/m^2/s red light, respectively. 5 FRL and 10 FRL denote 5 and 10 μmol/m^2/s far-red light, respectively. Bars=5 mm. (B and D) Quantitative analyses of hypocotyl length of seedlings of indicated lines in different red and far-red light fluence rates, respectively. Data are presented as means ± SD, n = 30.

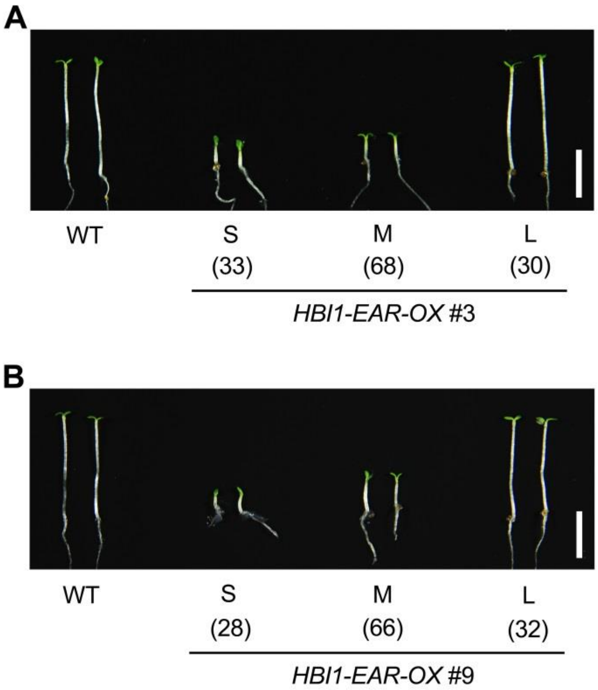

Supplementary Fig. S4. Phenotype analyses of siblings segregated from T2 heterozygous lines of *HBI1-EAR-OX*.

(A and B) Seedling phenotypes of indicated lines grown on half-strength MS medium under 2 μmol/m^2/s blue light for 5 days. Bars=5 mm. S, M, and L denote the segregated seedlings with short, medium and long hypocotyls, and the numbers of these seedlings were shown under these letters, respectively.

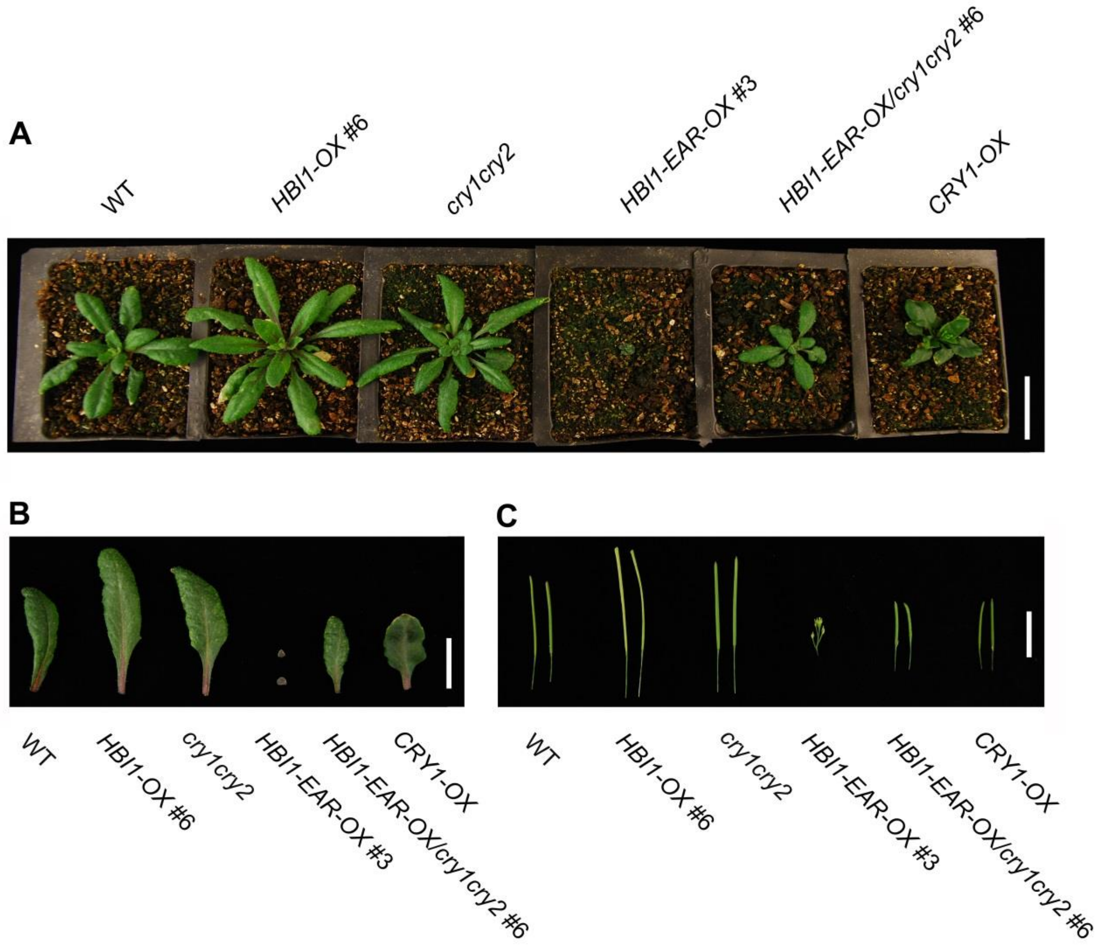

Supplementary Fig. S5. Phenotypic analyses of adult plants.

(A and B) Phenotypes of rosette and detached leaves with genotypes indicated grown in soil under continuous white light for 30 days. All the transgenic lines analyzed are homozygous. Bar=25 mm in (A) and 15 mm in (B). (C) Phenotypes of siliques with genotypes indicated grown in soil under continuous white light for 60 days. Bar=10 mm.

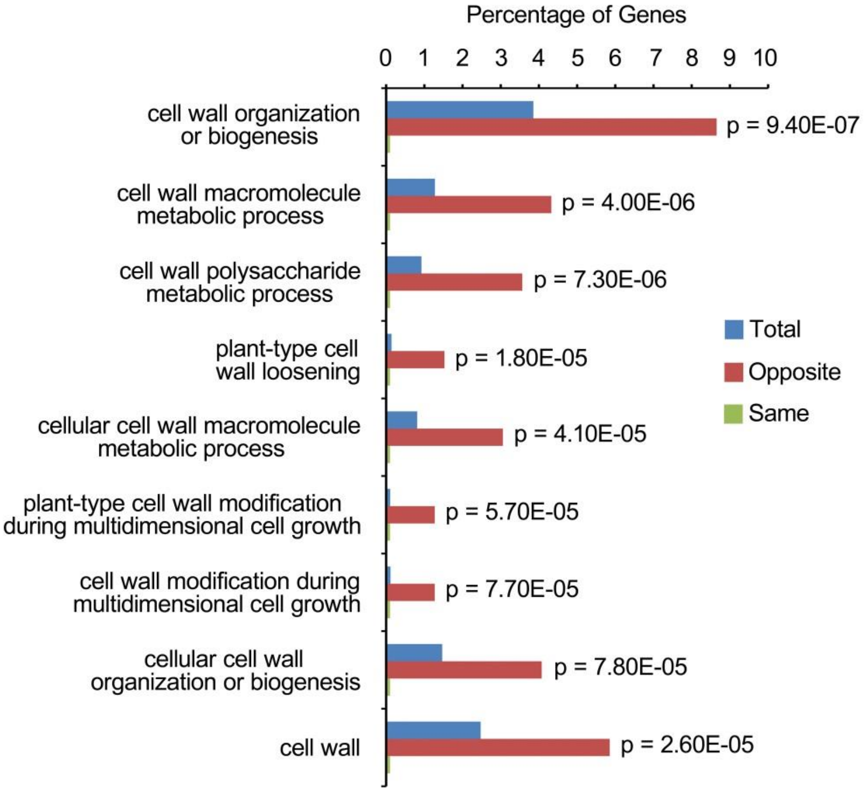

Supplementary Fig. S6. Gene Ontology (GO) analysis of overlapping genes regulated by HBI1 and CRYs.

"Total" denotes all genes in *Arabidopsis* genome; "Same" and "Opposite" denote genes regulated by HBI1 and CRYs in same and opposite direction, respectively. The p values were shown in each GO category.

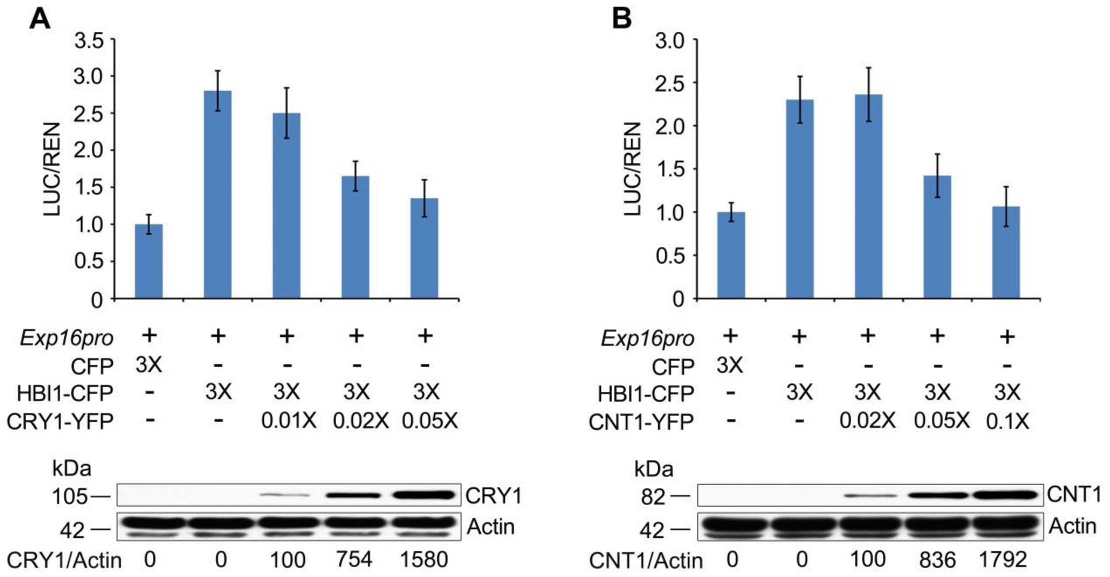

Supplementary Fig. S7. Dual-LUC assays showing CRY1 inhibition of the transcriptional activity of HBI1 through CNT1 in a dose-dependent manner.

(A and B) Tobacco leaves were transfected with *Agrobacterium* culture in different combinations of effectors and *Exp16*$_{pro}$ reporter and then kept in dim light for 3 days. 0.01×, 0.02×, 0.05×, 3× in (A) and 0.02×, 0.05×, 0.1×, 3× in (B) indicate the culture volume of effectors relative to that of *Exp16*$_{pro}$ reporter, respectively. The ratio of LUC activity relative to REN activity of expression control (CFP as effector) was arbitrarily set to 1, to which the ratios of other groups were normalized. Error bars represent ± SD (n=4). Corresponding expression levels of CRY1 (A) and CNT1 (B) shown in Western blot negatively correlate with measurements as a result of the extent of inhibitory effect of CRY1 and CNT1 on HBI1's transcriptional activity, respectively. Actin served as a loading control. CRY1/Actin (A) and CNT1/Actin (B) indicate the relative band intensities of CRY1 and CNT1 normalized to Actin and presented relative to that in combinations containing 0.01× CRY1-YFP and 0.02× CNT1-YFP set at unity, respectively.
